# Enhanced TLR7-induced interferon responses in women living with HIV

**DOI:** 10.3389/fimmu.2026.1791579

**Published:** 2026-04-21

**Authors:** Alisa Huber, Nadira Vadaq, Albert L. Groenendijk, Victoria Rios-Vazquez, Suzanne D.E. Ruijten, Rainer Knoll, Joost A.H. Martens, Adriana Navas, Vasiliki Matzaraki, Wilhelm A.J.W. Vos, Marc J.T. Blaauw, Louise E. van Eekeren, Maartje C.P. Jacobs-Cleophas, Anna C. Aschenbrenner, Joachim L. Schultze, Jan van Lunzen, Mihai G. Netea, Andre J.A.M. van der Ven, Leo A.B. Joosten, Jéssica C. dos Santos

**Affiliations:** 1 Department of Internal Medicine and Radboud Community for Infectious Diseases, Radboudumc, Nijmegen, Netherlands; 2 Department of Internal Medicine, ErasmusMC, Erasmus University, Rotterdam, Netherlands; 3 Department of Medical Microbiology and Infectious Diseases, ErasmusMC, Erasmus University, Rotterdam, Netherlands; 4 Systems Medicine, German Center for Neurodegenerative Diseases (DZNE), Bonn, Germany; 5 Department of Cell Biology, Faculty of Science, Radboud University, Nijmegen, Netherlands; 6 Department of Internal Medicine and Infectious Diseases, OLVG, Amsterdam, Netherlands; 7 Department of Internal Medicine and Infectious Diseases, Elizabeth-Tweesteden Ziekenhuis, Tilburg, Netherlands; 8 Genomics & Immunoregulation, Life & Medical Sciences Institute (LIMES), University of Bonn, Bonn, Germany; 9 Platform for single Cell genomics and Epigenomics (PRECISE), Deutsches Zentrum für Neurodegenerative Erkrankungen (DZNE)and University of Bonn and West German Genome Center, Bonn, Germany; 10 Department of Immunology and Metabolism, Life & Medical Sciences Institute (LIMES), University of Bonn, Bonn, Germany; 11 Department of Medical Genetics, Iuliu Hatieganu University of Medicine and Pharmacy, Cluj-Napoca, Romania

**Keywords:** HIV, HIV-control, interferon, sex-specific immunity, TLR7, viral suppression

## Abstract

**Introduction:**

Biological sex is a key modifier of HIV pathogenesis, with women more frequently achieving spontaneous viral control than men. Toll-like receptor 7 (TLR7), an endosomal RNA sensor encoded on the X chromosome that escapes X-inactivation, plays a pivotal role in antiviral immunity and is increasingly targeted in HIV cure strategies aimed at reversing viral latency. However, it remains unclear whether TLR7-driven immune responses differ by sex in the context of HIV infection.

**Methods:**

We characterized sex-specific immune responses to TLR7 stimulation in a cohort of 1,326 antiretroviral therapy (ART)-suppressed individuals living with HIV (192 women, 1,134 men), including 50 spontaneous HIV controllers, and in 43 people living without HIV (28 women, 15 men). Peripheral blood mononuclear cells (PBMCs) were stimulated ex vivo with the TLR7 agonist imiquimod (IMQ), followed by cytokine profiling and transcriptome analysis by RNA sequencing. To investigate transcriptional priming at baseline, we additionally analyzed single-cell RNA-sequencing (scRNA-seq) data from unstimulated PBMCs of 76 women and 214 men living with HIV.

**Results:**

PBMCs of women living with HIV (WLWH) released significantly lower amounts of IL-1β and MIP-1α (p < 0.01) following IMQ stimulation than PBMCs of men living with HIV (MLWH), with trends toward reduced IL-8 and IL-1Ra (p < 0.06), while IL-6 and MCP-1 production was similar across sexes. Transcriptomic analysis revealed sex-dependent gene programs following TLR7 activation. Women living without HIV (WLWoH) showed selectively higher IFNγ (type II interferon) signaling and downregulated B cell-associated transcripts compared to men living without HIV (MLWoH). In contrast, WLWH exhibited a pronounced induction of both IFNα (type I) and IFNγ (type II) pathways, marked by elevated expression of interferon-stimulated genes including IRF7, ISG15, MX1, and APOBEC3A, alongside reduced antibacterial and inflammatory signatures compared to MLWH. Single-cell RNA sequencing further identified IRF7 as a key ISG selectively upregulated in plasmacytoid dendritic cells (pDCs) of WLWH. These transcriptional responses were independent of pDC frequency and did not differ between HIV controllers and non-controllers.

**Discussion:**

Despite the male-biased cohort, these findings demonstrate that women mount stronger interferon-driven responses upon TLR7 activation compared to men, accompanied by attenuated inflammatory cytokine production. These sex-based immunological differences may contribute to improved viral control in women and highlight the importance of incorporating sex as a biological variable in TLR7-targeted HIV immunotherapies.

## 1 Introduction

Human immunodeficiency virus type 1 (HIV-1; hereafter referred to as HIV) remains a major global health challenge, with over 38 million people living with HIV (PLWH) worldwide (“^1^). Although antiretroviral therapy (ART) has transformed HIV into a manageable chronic condition for most individuals (2), the virus persists in a latent reservoir of transcriptionally silent, long-lived infected cells that remains impervious to current treatments (3–6). Eradicating this reservoir and reversing immune dysregulation continue to be primary goals in the search for a functional cure (7).

Among PLWH, biological sex is a critical but often underappreciated determinant of immune responses and clinical outcome. Women (defined here as cisgender women, with men denoting cisgender men) account for over half of the global PLWH population, and numerous studies have revealed sex-based disparities in HIV pathogenesis (8–11). Women tend to exhibit lower plasma viral loads and a smaller viral reservoir than men at comparable stages of infection (8, 11). Moreover, spontaneous viral control in the absence of ART is disproportionately observed in women, with odds ratios ranging from 1.9 to 5 in large cohort studies (12, 13). These differences persist even when adjusting for sociodemographic factors and are thought to arise, at least in part, from intrinsic immunological differences between the sexes (13).

While sex steroid hormones (e.g., estrogen, progesterone, and testosterone) can modulate immune cell function and cytokine production (14–16), recent attention has turned to the role of sex chromosome-linked immune regulators, particularly the viral receptor Toll-like receptor 7 (TLR7). TLR7 is a pattern recognition receptor encoded on the X chromosome that senses single-stranded RNA (ssRNA) viruses, including HIV (17). Upon ssRNA recognition, TLR7 initiates signaling cascades via MyD88 and IRAK4, activating NF-κB and interferon regulatory factors (IRFs) that promote the expression of pro-inflammatory cytokines such as IL-6 and TNF, as well as type I interferons (IFN-α and IFN-β) (18–20).

Importantly, TLR7 is among the subset of X-linked genes that can escape X-chromosome inactivation (XCI) in female immune cells, such as plasmacytoid dendritic cells (pDCs) and B-cells, (21) leading to biallelic expression and elevated TLR7 mRNA and protein expression in immune cells of women compared to men (22, 23). Estrogen further amplifies this response by enhancing both TLR7 expression and downstream signaling (24). In the context of HIV, elevated TLR7-driven responses in women may initially confer an advantage by promoting rapid antiviral responses that contain viral replication (25, 26).

Enhanced IFN-α production by pDCs upon TLR7 stimulation has been observed in women and is proposed to underlie the enhanced initial antiviral response towards HIV (27). While early and robust IFN-I responses can contribute to initial control of viral replication, sustained activation may drive immune dysregulation over time (28, 29). In the context of HIV, elevated type-I interferon responses - particularly in women - have been linked to increased immune activation and altered T cell function (30) (31, 32). In PLWH, patterns of chronic immune activation and inflammatory dysregulation, particularly among women, have been associated with elevated risk for non-AIDS complications, including cardiovascular disease, neurocognitive impairment, and autoimmune manifestations (33, 34).

This ambivalent role of interferons underscores the need to understand the regulation of TLR7 signaling in PLWH, especially considering its sex-biased expression and potential implications for therapeutic targeting. Despite this growing body of evidence, it remains unclear how TLR7 responses are shaped by chronic HIV infection, how they differ between women and men, and whether enhanced TLR7-driven immunity is associated with spontaneous HIV control or represents an intrinsic sex-biased program. To address these questions, we examined *ex-vivo* cytokine and transcriptomic responses to TLR7 stimulation in PBMCs of ART-suppressed PLWH, including individuals who spontaneously control the virus without ART (so-called HIV controllers), as well as in HIV-seronegative individuals.

Our findings reveal that in the context of HIV infection, TLR7-driven immune responses differ substantially between sexes, with women exhibiting reduced inflammatory cytokine production but enhanced type I interferon-associated transcriptional activity compared to men, highlighting the need for sex-informed approaches in HIV immunotherapy.

## 2 Materials and Methods

### 2.1 Cohort

We enrolled 1895 PLWH from October 2019 until October 2021 as part of the 2000HIV study (NCT03994835). This large cohort study is composed of participants from four specialized Dutch HIV treatment centers, two university medical centers and two large general hospitals. Participants were allocated either to a discovery cohort or an independent validation cohort. Detailed characteristics of this multiomics approach have been described elsewhere (35). Spontaneous HIV controllers (HIC) were classified as 1. elite controllers (ECs) with HIV-1 RNA <75 copies/mL for more than 12 months in the absence of cART with stable CD4 T cell counts (> 500 cells/mm^3^); 2. viremic controllers (VC) characterized by HIV-1 RNA <10.000 copies/mL for at least 5 years in the absence of cART with stable CD4 T cell counts and 3. transient controllers, who exhibited plasma HIV-RNA levels exceeding 10,000 copies/mL after initially meeting the criteria for being classified as an HIV controller. ART was initiated in a subset of HIV controllers during follow-up due to factors, such as transmission prevention, patient preference or adherence to new HIV treatment guidelines introduced in 2015, which recommended ART for all PLWH regardless of viral load levels or CD4 counts. Both ART-naïve and ART-exposed ECs, VCs, and transient controllers are summarized as HIC in this study. All study participants provided written informed consent. Due to the voluntary registration of most PLWH in the Netherlands in a national cohort (ATHENA cohort, Stichting HIV Monitoring), a full overview of the general and HIV-related medical history of our participants was obtained. Hematocytometry measurement and *ex vivo* cytokine data were analyzed from 1326 PLWH from the discovery cohort. The reference to the sex of participants is based on sex at birth and is not based on the self-identified gender of participants. Individuals who self-identified as transgender and had a recorded use of hormonal replacement therapy were excluded from the analysis, as hormonal usage might have confounded the results. Classification of women pre-, peri or post-menopause were based on questionnaire answers of the participants but were confirmed by measuring follicle-stimulating hormone (FSHB) concentrations in plasma. The ethnicity of participants was self-defined in the questionnaire. The 2000HIV was approved by the Medical Ethical Review Committee Oost Nederland, Nijmegen, the Netherlands NL68056.091.81 and published at clinicaltrials.gov.

HIV-seronegative controls (n=43) were obtained from the 2000HIV-TRAINED sub-study (NCT04968717), that also enrolled 1st-degree family members of PLWH. Inclusion and exclusion criteria of the 2000HIV-TRAINED sub-study, previously described by us (36).

### 2.2 Hemocytometric measurement

Whole blood of 1326 PLWH (1134 men and 192 women) was collected during daytime hours at the participating centers and sent overnight to the coordinating center at Radboudumc. The overnight shipping was at room temperature, and samples were processed immediately in the morning, the day after the baseline visit. The samples collected in the coordinating center, were also stored overnight at room temperature to ensure identical handling across all centers. Upon arrival of the samples in the lab, whole blood obtained from EDTA tubes was used for hemocytometry measurement with the XN Sysmex hematology analyzer (Sysmex Corporation, Kobe, Japan).

### 2.3 *Ex vivo* cytokine production assay

Human peripheral blood mononuclear cells (PBMCs) of 1326 PLWH (1134 men and 192 women) were obtained from venous blood collected in EDTA tubes and were isolated by density centrifugation of blood diluted 1:1 in pyrogen-free phosphate-buffered saline (PBS) over Ficoll-Paque (GE Healthcare, Chicago, IL, USA). PBMCs were seeded at 500,000 cells/well in U-bottom plates (Corning, Corning, NY, USA) and subsequently stimulated with 5 µg/mL of the TLR7 agonist imiquimod (IMQ; Invivogen, San Diego, CA, USA) for 24 hours at 37 °C and 5% CO2. The production of cytokines and chemokines (IL-1β, IL-1Ra, IL-6, IL-8, MCP-1, and MIP-1a) were assessed in the supernatants by ELISA (R&D Systems, Minneapolis, MN, USA). Additional measurements were performed for PBMCs of WLWH (n=18) and MLWH (n=41) of the 2000HIV-TRAINED study using an Ella Automated Immunoassay System (BioTechne, Minneapolis, MN, USA) according to the manufacturer’s recommendations. For the subselection IFNα and IFNγ were measured after stimulation with 5 µg/mL (IMQ; Invivogen, San Diego, CA, USA)for 24 hours at 37 °C and 5% CO_2_.

### 2.4 Single-cell RNA sequencing

#### 2.4.1 Single-cell RNA sequencing using BD rhapsody

Cryopreserved PBMCs of n=76 women and n=214 men with HIV were thawed in a 37 °C water bath, diluted in pre-warmed RPMI-1640 medium supplemented with 10% FBS, and centrifuged at 300 g for 5 min. After washing, cells were resuspended in RPMI-1640 + 10% FBS and prepared for single-cell transcriptomics using the BD Rhapsody Single-Cell Analysis System (BD Biosciences, San Jose, CA, USA).

Samples were individually labelled with hashtag-oligonucleotide-conjugated antibodies (BD Human Single-Cell Multiplexing Kit) according to the manufacturer’s protocol, pooled in equal ratios, and stained with the BD AbSeq Immune Discovery Panel. After Fc blocking and antibody incubation, cells were washed, counted, and loaded onto BD Rhapsody cartridges (60,000 cells per cartridge) for single-cell capture and cDNA synthesis.

Libraries were prepared using the BD Rhapsody Whole Transcriptome Analysis (WTA) Amplification Kit and Sample Tag/AbSeq Library Prep protocols. Final library quality was assessed using a Qubit fluorometer and TapeStation 4200 (Agilent). Sequencing was performed on an Illumina NovaSeq 6000 (paired-end, 2 × 75 cycles) using S2 or S4 chemistry.

#### 2.4.2 Preprocessing and alignment of scRNA-seq data

Demultiplexing of raw BCL files was performed with bcl2fastq2 (v2.20). Adapter trimming and quality filtering (PHRED ≥20) were done using cutadapt (v1.16). Reads were aligned to the human genome (GENCODE v33) using STAR (v2.7.3a). Drop-seq tools (v2.0.0) were used for UMI quantification and generation of digital expression matrices. Hashtag oligo and AbSeq tag sequences were appended to the reference genome for multiplexed sample demultiplexing.

#### 2.4.3 Quality control, normalization, and clustering

Data processing was performed in Seurat (v4.3.0). Cells were retained if they were singlets (identified via HTODemux and vireo), expressed >500 UMIs, contained <35% mitochondrial reads and <5% haemoglobin transcripts, and had between 250 and 2500 detected genes. Contaminating or low-quality clusters were excluded. Genes expressed in fewer than five cells per cartridge were removed.

Gene expression data were normalized using total UMI counts per cell, scaled, and log-transformed. Dimensionality reduction was performed on the top 2,000 variable genes using PCA, followed by Harmony integration to account for CENTER, SEASON, and SEX_BIRTH effects.

A Weighted Nearest Neighbor (WNN) approach was used to integrate RNA and AbSeq data. UMAP was computed on the multimodal WNN space (min.dist = 0.1, n.neighbors = 50), and clustering was performed using the SLM algorithm at multiple resolutions. Marker genes were identified via Wilcoxon rank sum test (FindAllMarkers, min.pct = 0.2, logfc.threshold = 0.5). Clusters were annotated based on marker expression and literature-derived signatures. Major cell types were further subclustered and annotated in an iterative process.

### 2.5 Phenotyping of circulating pDCs using flow cytometry

Whole blood was obtained from venous blood collected in EDTA tubes, and pDCs were assessed by flow cytometry as previously described by Navas et al. (37). In short, for this study, we used custom-made DuraClone tubes by Beckman Coulter (Beckman Coulter Life Sciences, Indianapolis, IN, USA). **Supplementary Table 1** shows the information for all antibodies included in Panel 1, that were either drop-in antibodies or pre-coated in the DruaClone tubes. Following exclusion of granulocytes and monocytes, CD3^–^ cells were identified, from which total dendritic cells were defined as HLA-DR+CD19^–^. Within this DC gate, pDCs were classified as CD123+CD11c^–^ and myeloid DCs as CD123^–^CD11c+, with further mDC subclassification based on CD16 and CD1c expression, as previously described. pDC frequencies were assessed in 1,115 MLWH and 226 WLWH.

### 2.6 High-throughput transcriptomics (bulk PBMCs RNA sequencing)

#### 2.6.1 Preprocessing

Bulk RNA sequencing of PBMCs after stimulation with IMQ and at baseline RPMI condition was performed for a selection of the 2000HIV cohort, consisting of n=27 women and n=43 men living with HIV, as well as n=28 women and n=15 men living without HIV, following detailed protocols (35, 38). The selection included 28 HIC (14 of which were ART-naïve ECs) and 35 non-HIC. Initial quality control procedures involved the exclusion of samples lacking information on uniquely mapped reads, duplicate entries, those with fewer than five million uniquely mapped reads, and discrepancies in biological sex and chr-y expression. Quality control ensured robust data for subsequent analyses.

#### 2.6.2 Differential expression analysis

Differential expression analysis (DEA) was performed to investigate RNA expression patterns in PBMCs from comparison groups (for comparisons, see the results section). The DESeq2 workflow (38b) was used for transcriptomics data, applying negative binomial generalized linear models, followed by multiple testing correction using Independent Hypothesis Weighting (IHW), and Log2 fold change (Log2FC) shrinkage via the “apeglm” method. The analysis was adjusted for confounders, based on principal component analysis and differences between groups (for precise confounder selection, see results). Differentially expressed genes (DEGs) were identified based on a false discovery rate (FDR) threshold of < 0.05 and a Log2FC of +/-1.5.

#### 2.6.3 Gene set enrichment analysis

Pathway enrichment analysis was performed to identify pathways significantly associated with transcriptomic markers of participants between women and men, using pathway enrichment analysis on ranked gene lists derived from differential expression results. A combined score, calculated as -Log10(P-value)×sign(Log2FC), was used to rank genes based on comparison, capturing both the significance and direction of expression changes. These ranked gene lists were then analyzed across multiple pathway databases using the clusterProfiler package (39). Enrichment analyses included Hallmark gene sets, Kyoto Encyclopedia of Genes and Genomes (KEGG) pathways, Gene Ontology (GO) biological process (BP), molecular function (MF), and cellular component (CC) ontologies, as well as Reactome, WikiPathways, and Disease Ontology (DO) databases (40–44). For each analysis, enrichment scores were calculated to assess pathway significance, applying an adjusted p-value cutoff of 0.05 and using the Benjamini-Hochberg (BH) method for correction of multiple testing. This comprehensive approach allowed enriched pathways to be identified, offering insights into the biological mechanisms underlying transcriptomic differences between groups.

### 2.7 Measurement of FSHB concentrations in plasma

Plasma proteomics was conducted using the Olink^®^ Explore 3072 panel, which employs a proximity extension assay in which proteins bind to oligonucleotide-labelled antibodies, as detailed in previous literature (45, 46). These sequences are amplified and quantified by qPCR or NGS, reflecting protein concentrations. FSHB concentrations were compared among women who self-reported to be in pre-, peri-, or post-menopause.

### 2.8 Statistical analysis

Group comparisons (**Tables 1**, **2**) were performed using appropriate statistical tests based on the type and distribution of variables. Continuous variables were compared using a Wilcoxon rank-sum test to assess means between two groups, taking into account potential unequal variances. Categorical variables were compared with the Chi-square test when expected cell counts were sufficient (≥5). Fisher’s exact test was utilized for categorical comparisons when sample sizes were small or expected frequencies in any cell fell below 5. Statistical significance was set at p < 0.05. Visualizations and statistical tests were performed in R 4.3.1 (RStudio, PBC, Boston, MA, USA).

**Table 1:** Baseline characteristics between women and men of the 2000HIV cohort used for ex vivo cytokine production assay.

	Sex at birth		
Variable	Men living with HIV N = 1,134^1^	Women living with HIV N = 192^1^	p-value^2^
**Age in years**	52 (12)	51 (13)	0.12
**BMI**	25.1 (3.8)	27.1 (5.9)	<0.001
**Ancestry**			<0.001
Asian	55 (4.9%)	9 (4.7%)	
Black	78 (6.9%)	62 (32%)	
Hispanic	35 (3.1%)	5 (2.6%)	
Mixed	79 (7.0%)	20 (10%)	
Native American	1 (<0.1%)	0 (0%)	
White	886 (78%)	96 (50%)	
**HIV Acquisition Route**			<0.001
Blood products	3 (0.3%)	1 (0.6%)	
Congenital	7 (0.6%)	1 (0.6%)	
Heterosexual	92 (8.5%)	170 (94%)	
IV drug use	9 (0.8%)	8 (4.4%)	
MSM	972 (90%)	0 (0%)	
Unknown	51	12	
**HIV Duration in years**	13 (8)	16 (8)	<0.001
**cART Duration in years**	11 (7)	14 (7)	<0.001
Unknown	15	9	
**CD4 Nadir**	0.30 (0.22)	0.26 (0.26)	0.002
Unknown	17	5	
**VL Latest Measurable**			0.8
Undetectable^3^	1,100 (97%)	185 (96%)	
Detectable^4^	34 (3.0%)	7 (3.6%)	
**VL latest in copies/mL** **(for detectable means)**	71 (68)	205 (367)	0.078
**VL Zenith**	603,390 (3,423,316)	414,542 (1,339,439)	0.010
Unknown	90	23	
**Inclusion Center**			0.009
EMC	432 (38%)	56 (29%)	
OLV	514 (45%)	89 (46%)	
RUMC	188 (17%)	47 (24%)	
**Inclusion Season**			0.018
Autumn	276 (24%)	61 (32%)	
Spring	163 (14%)	35 (18%)	
Summer	402 (35%)	49 (26%)	
Winter	293 (26%)	47 (24%)	
**Inclusion before or after COVID-19 pandemic**			<0.001
Before	237 (21%)	13 (6.8%)	
After	897 (79%)	179 (93%)	
**Infection with SARS-CoV-2**	129 (11%)	33 (17%)	0.031
**Vaccination against SARS-CoV-2**	286 (25%)	56 (29%)	0.3
**Infection with HCMV**	1066 (94%)	179 (93%)	0.7
**Infection with HCV**	116 (10%)	13 (6.8%)	0.2
**HIV Controller Status**			0.009
HIC	36 (3.2%)	14 (7.6%)	
Non-HIC	1,073 (97%)	171 (92%)	
Unknown	25	7	
**HIV Controller Type**			0.011
EC (no ART)	12 (1.1%)	7 (3.9%)	
EC (ART)	3 (0.3%)	4 (2.1%)	
VC (ART)	21 (1.9%)	3 (1.7%)	

^1^Median (IQR) for continuous; n (%) for categorical.

^2^Wilcoxon rank sum test for continuous; Pearson’s Chi-squared test (or Fisher’s exact test if n < 5) for categorical.

^3^Unmeasurable, unquantifiable or <40 copies/mL.

^4^>40 copies/mL, exact quantification.

cART, combination antiretroviral therapy; HCMV, Human cytomegalovirus; HCV, hepatitis C virus; HIC, HIV controllers; Non-HIC, normal progressors PLWH; EC, elite controllers; VC, viraemic controllers; ART, Anti-Retroviral Therapy. Bold text indicates category headers; non-bold entries represent subcategories within each variable.

**Table 2:** Clinical and inclusion factors between women and men of a subselection of individuals from the 2000HIV cohort used for bulk-transcriptomics analysis.

	Sex at birth		
Variable	Female N = 27^1^	Male N = 43^1^	p-value^2^
**AGE**	45 (9)	52 (13)	0.009
**BMI BASELINE**	26.6 (6.6)	26.2 (3.8)	0.8
**CENTER**			0.063
EMC	6 (22%)	16 (37%)	
ETZ	3 (11%)	3 (7.0%)	
OLV	9 (33%)	20 (47%)	
RUMC	9 (33%)	4 (9.3%)	
**ETHNICITY**			<0.001
Asian	1 (3.7%)	1 (2.3%)	
Black	12 (44%)	2 (4.7%)	
Hispanic	1 (3.7%)	0 (0%)	
Mixed14	2 (7.4%)	5 (12%)	
White	11 (41%)	35 (81%)	
**SEASON**			0.6
Autumn	10 (37%)	11 (26%)	
Spring	8 (30%)	13 (30%)	
Summer	9 (33%)	19 (44%)	
**COVID19**	9 (33%)Figure	10 (23%)	0.4
**COVID19 VACCINE**	11 (41%)	31 (72%)	0.013
**CONTROLLER STATUS**			>0.9
HIC	14 (52%)	21 (49%)	
Non-HIC	13 (48%)	22 (51%)	

^1^Mean (SD); n (%).

^2^Wilcoxon rank-sum test for continuous; Chi-square test for categorical variables (Fisher’s exact test was used when expected counts were <5). bold text indicates category headers; non-bold entries represent subcategories within each variable.

To test for differences in cytokines and chemokines across the different PLWH groups, a rank-based regression model with selected confounders was used in R 4.3.1 (RStudio, PBC, Boston, MA, USA). Seasonality as a confounder was modelled using a sine and cosine wave with a period of 365.25 days as previously described (47). Together, these two terms can form a sine wave with any phase with a frequency of one year. The relative percentages of pDCs between n=226 WLWH and n=1115 MLWH were normalized using an inverse rank transformation and compared with a linear regression model with selected confounders. All resulting p-values were corrected for multiple testing (FDR).

To identify potential confounders, we assessed associations between the first five PCs as calculated using the data of interest and potential confounders using a linear regression model. The first five genetic PCs were assessed to correct for ancestry. We selected as potential confounders those that showed a beta coefficient > 0.05 from a linear regression on the first five PCs. The selected confounders for each comparison are indicated in the appropriate result section and in **Supplementary Figure 1**.

## 3 Results

### 3.1 Cohort characteristics reveal demographic and clinical differences between women and men with HIV

To investigate sex-based differences in immune responses to TLR7 stimulation, we included 1,134 men and 192 women from the 2000HIV discovery cohort. A small proportion of participants were spontaneous HIV controllers (HICs), comprising 3.2% of men and 7.6% of women, p = 0.009). The median age was comparable between MLWH and WLWH in the full cytokine cohort (52 vs 51 years, p=0.12).

Key demographic and clinical characteristics differed significantly between sexes (**Table 1**). Women exhibited a higher mean body mass index (BMI) than men (27.1 vs. 25.1, p < 0.001). Ethnicity also varied substantially: while the majority of men were of European descent (78%), women were more ethnically diverse, with only 50% identified as European and 32% as having a sub-Saharan African ancestry (p < 0.001). Given the documented impact of ethnicity on immune responses and cytokine production, this variable was considered in downstream analyses.

The route of HIV acquisition diverged markedly between sexes. Most men identified as men who have sex with men (MSM; 90%), whereas women predominantly acquired HIV via heterosexual contact (94%, p < 0.001). A minor proportion of women reported intravenous drug use (4.4% vs. 0.8% in men, (p < 0.001). Duration of HIV infection and ART use were longer in women than in men (HIV duration: 16 vs. 13 years; ART duration: 14 vs. 11 years; both p < 0.001), which could influence immune reconstitution and long-term immune activation. Nadir CD4 counts were slightly lower in women (0.26 vs. 0.30 ×10^9^/L, p = 0.002), although the proportion of immunological non-responders was comparable. There was no difference in recent viral load detection (p=0.8).

Other potentially confounding variables also differed by sex. More women were enrolled after the onset of the COVID-19 pandemic (93% vs. 79%, p < 0.001), and more often reported previous SARS-CoV-2 infection (17% vs. 11%, p = 0.031). There were no significant differences in SARS-CoV-2 vaccination status (p = 0.3). Human cytomegalovirus (HCMV) is a common co-infection in PLWH (48, 49), which was confirmed in our cohort, as 94% of men and 93% of women living with HIV were seropositive for HCMV, not differing between sexes. While intravenous drug use was a prevalent route of HIV acquisition among women and is closely linked to Hepatitis C Virus (HCV) infection (50, 51), there was no observed difference in HCV positivity between women and men with HIV.

Together, these data illustrate meaningful demographic and clinical differences between men and women in the cohort that were explored and partly used as confounding variables in downstream analysis if applicable.

### 3.2 Women living with HIV produce lower amounts of inflammatory cytokines and chemokines upon IMQ stimulation compared to men living with HIV

**Figure 1:**
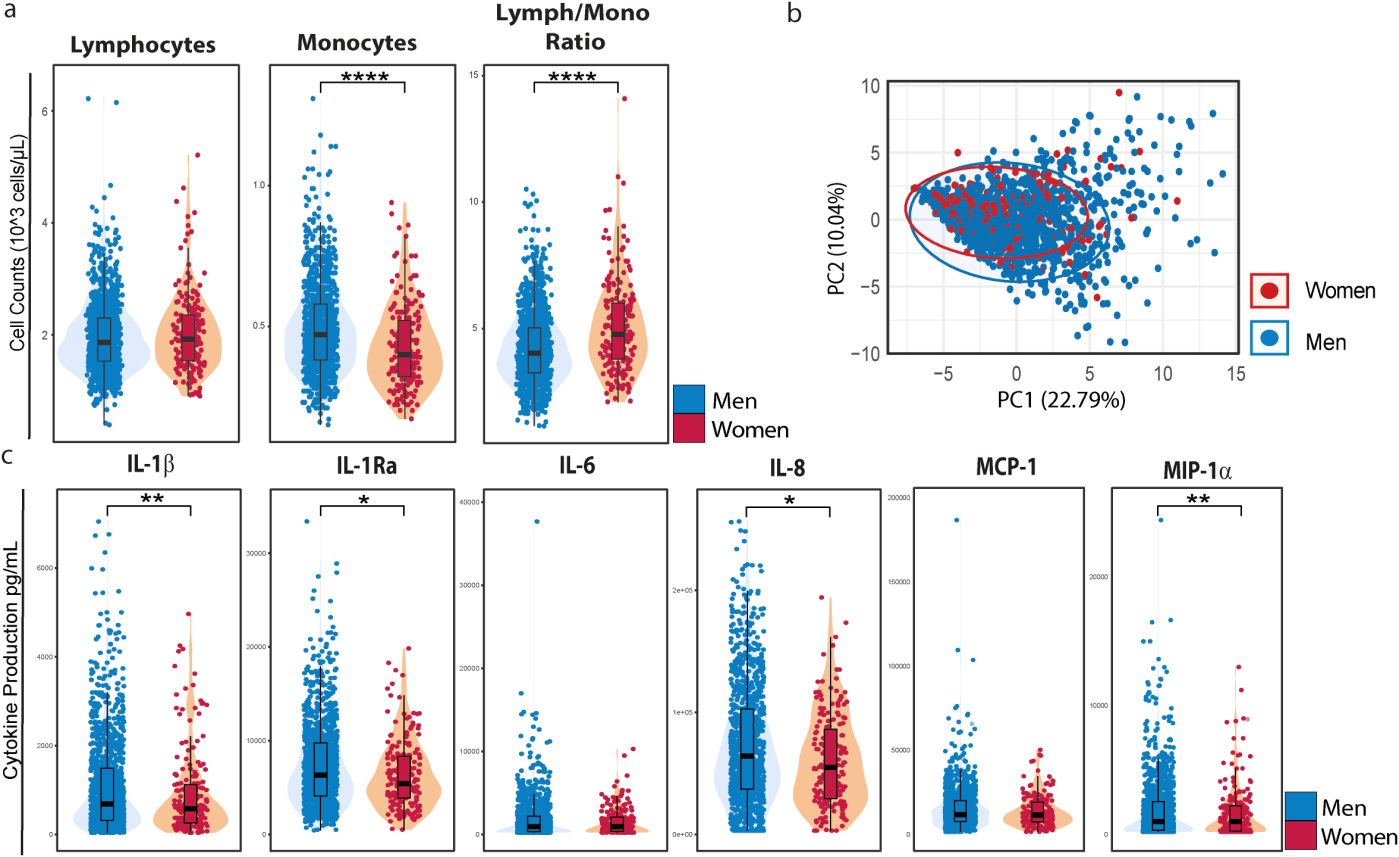
PBMCs Of WLWH produce less inflammatory cytokines compared to PBMCs of MLWH Upon TLR7 activation. **(a)** Whole blood of 192 women living with HIV and 1134 men living with HIV was used to measure circulating immune cell distribution. Counts of monocytes and lymphocytes as well as the lymphocyte/monocyte Ratio between women and men living with HIV. Significance was tested using a Wilcoxon rank-sum test **(b)** PCA plot of cytokine production values in women and men. **(c)** PBMCs were isolated and stimulated with IMQ for 24 hours. Cytokines between women and men living with HIV were measured in the supernatant. Significance was tested using a logistic regression model, adjusted for age, season of inclusion, inclusion before or after COVID-19 pandemic, ancestry, and lymphocyte/monocyte ratio. P-values adjusted for multiple testing. Asterisks indicate statistical significance: *P < 0.05, **P < 0.01, ***P < 0.001, ****P < 0.0001.

As differences in immune cell counts and percentages can influence overall immune responses, we first assessed differences in immune cell composition between women and men. We measured lymphocyte and monocyte numbers in whole blood of 1,134 men living with HIV (MLWH) and 192 women living with HIV (WLWH) using a hematology analyzer. While WLWH had comparable numbers of lymphocytes as MLWH, WLWH showed reduced numbers of circulating monocytes (p = 2.6 x10^-8^, **Figure 1a**). The lymphocyte/monocyte ratio was calculated using the total number of cells and was consequently higher in WLWH compared to MLWH (p= 1.3x10^-8^). Eosinophil and basophil counts were also reduced in WLWH, but neutrophil counts were comparable between MLWH and WLWH (**Supplementary Figure 1a**).

We next assessed whether the inflammatory cytokine responses of PBMCs upon IMQ stimulation differ between WLWH and MLWH. As differences in immune cell composition can influence cytokine responses, our data were corrected for the lymphocyte/monocyte ratio to avoid confounding by lower monocyte numbers in women. Principal component analysis (PCA) revealed that sex was a strong driver of cytokine production capacity (**Figure 1b**). Upon 24 hours IMQ stimulation, PBMCs from WLWH produced significantly lower amounts of IL-1β (p=0.0009), MIP1α (p=0.0051), IL-8 (p=0.0275) and IL-1RA (p=0.0187) compared to MLWH. There was no significant difference of IL-6 and MCP-1 production in WLWH compared to MLWH (**Figure 1c**).

In addition, we also measured type-I (IFNα) and type-II (IFNγ) interferon responses to TLR7 stimulation in a subset of the study participants. IFNα levels were low in the cell supernatant, as all values were below the detection limit of the assay. As for IFNγ, we did not find any differences in the production by PBMCs of 18 WLWH compared 41 MLHW (**Supplementary Figure 1c**).

### 3.3 pDCs of women living with HIV are primed with higher baseline expression of IRF7

**Figure 2:**
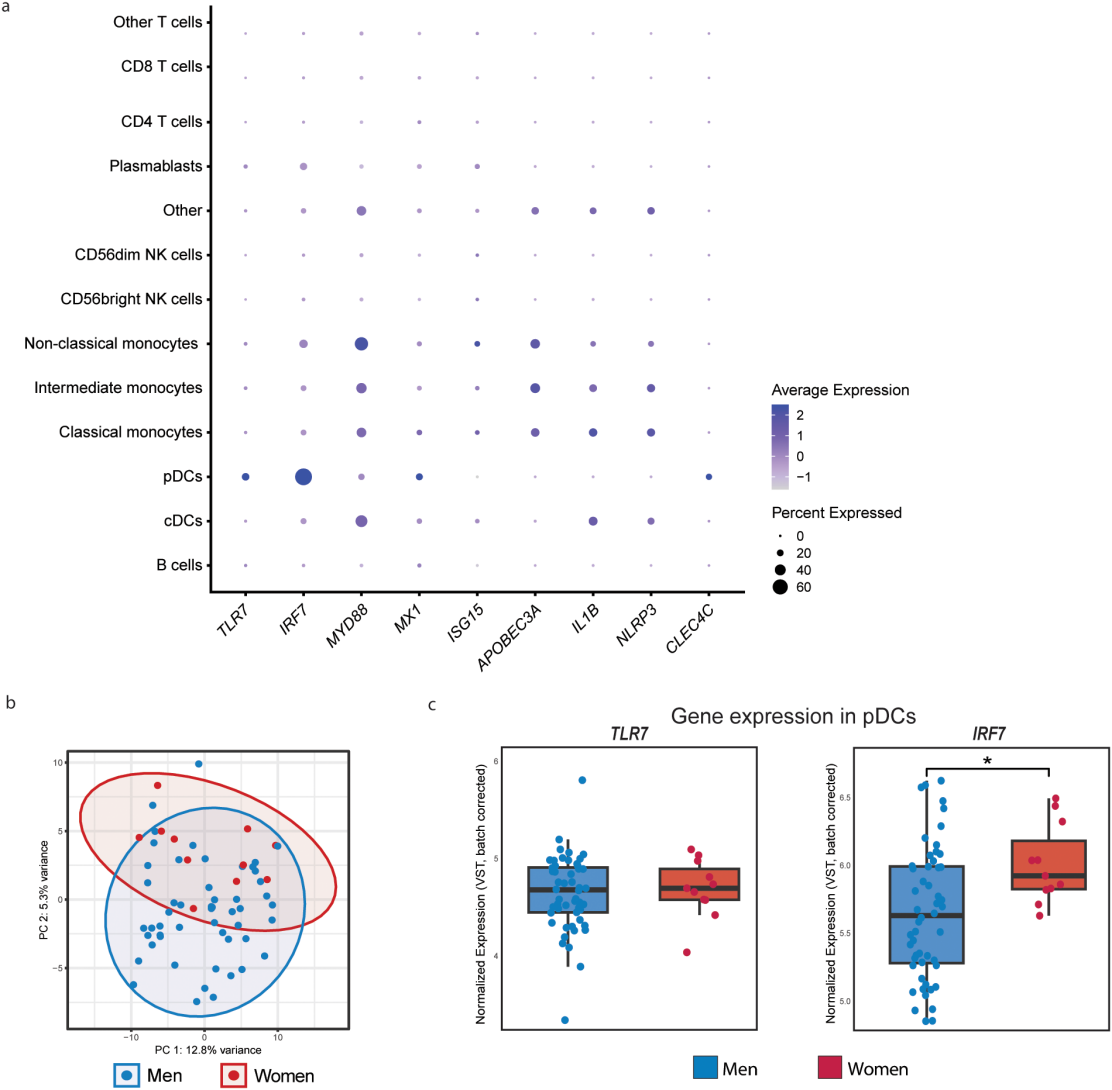
Baseline single-cell transcriptomics reveals sex-biased IRF7 expression in pDCs Of PLWH. **(a)** Dot plot depicting the relative expression and proportion of cells expressing selected genes related to TLR7 signaling, type I IFN responses, and inflammation across immune cell types. **(b)** Principal component analysis (PCA) of pseudobulked gene expression profiles from pDCs of women (n = 11) and men (n = 52) living with HIV. Percent variance explained by PC1 and PC2 is indicated. **(c)** Box plots showing batch-corrected normalized expression of TLR7 and IRF7 in pDCs, comparing women and men living with HIV. Pseudobulked data of pDCs was adjusted for Age, center of inclusion, inclusion before or after the COVID-19 pandemic, and vaccination against SARS-CoV-2. Significance was tested using a Wilcoxon rank-sum test. Asterisks indicate statistical significance: *P < 0.05.

To investigate transcriptional programming at baseline, we analyzed scRNA-seq data of baseline PBMCs of n = 76 WLWH and n = 214 MLWH. All expected major immune cell subsets were identified using literature-validated marker genes, including T cells, pDCs, monocytes, B cells, and NK cells (**Supplementary Figures 2a, b**). Guided by our cytokine findings and the known biology of TLR7 signaling, we first assessed the expression of genes representing key pathways across all immune cell subsets. These included genes involved in TLR7 activation and signaling (*TLR7, IRF7*, *MYD88*), interferon responses (*MX1, ISG15*), antiviral restriction (*APOBEC3A*), inflammatory mediators corresponding to cytokines we measured (*IL1B, NLRP3*), and pDC identity and function (*CLEC4C*) (**Figure 2a**). Among these, *IRF7* stood out for its strong and cell type–specific expression, being strongly expressed in pDCs. Supporting our cell type annotation, TLR7 was also mainly expressed in pDCs, while *IL1B* and *NLRP3* were enriched in monocytes.

To quantify this observation, we pseudobulked the data by summing counts across pDCs (min. 20 cells/donor) from each donor, yielding in data from 11 WLWH and 52 MLWH. PCA analysis revealed that sex was a major driver of gene expression in pDCs (**Figure 2b**). While *TLR7* expression did not significantly differ between the sexes, *IRF7* expression was significantly higher in MLWH than in MLWH (p = 0.019; **Figure 2c**).

To rule out confounding by pDC abundance, we assessed pDC frequency and activation status via whole-blood flow cytometry in an expanded PLWH cohort (MLWH n = 1,115; WLWH n = 226). No significant sex differences were found in pDC percentages (**Supplementary Figure S3a, b**), nor between HIC and non-HIC (FDR = 0.208, **Supplementary Figure 3c**) Together, these analyses indicate that IRF7 is specifically enriched in pDCs and is expressed at higher levels in women living with HIV compared to men living with HIV. The selective upregulation of IRF7 in pDCs of WLWH at baseline suggests enhanced priming of the TLR7-MyD88-IRF7 signaling axis, predisposing these cells to stronger downstream ISG induction upon TLR7 activation.

### 3.4 Sex drives transcriptomic responses to TLR7 stimulation in PBMCs of people living with or without HIV

Given the elevated baseline expression of *IRF7* in pDCs of WLWH and the sex-specific cytokine responses observed upon TLR7 stimulation, we next asked whether this transcriptional priming translates into broader differences in gene expression. To address this, we analyzed bulk transcriptomic profiles of PBMCs following IMQ exposure. To distinguish responses intrinsic to sex from those shaped by chronic HIV infection, we performed bulk transcriptomic profiling of PBMCs from both PLWH (n=27 WLWH; n=43 MLWH, **Table 2**) and people living without HIV (PLWoH) after stimulation with the TLR7 agonist imiquimod (IMQ).

**Figure 3:**
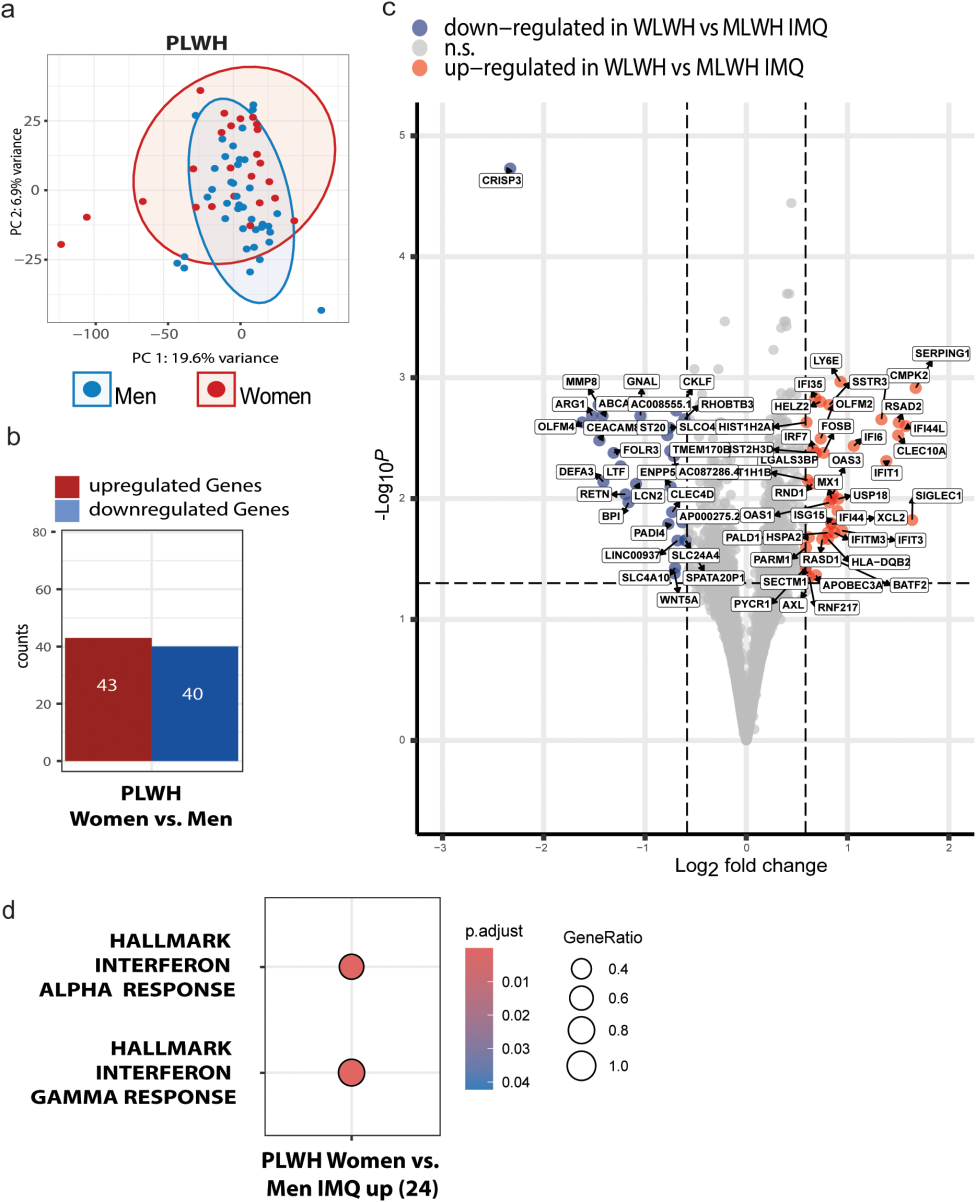
Differential gene expression and pathway enrichment in TLR7−stimulated PBMCs highlight augmented type I/II interferon signatures in WLWH. **(a)** PBMCs of 27 women and 43 men living with HIV were stimulated with IMQ for 24 hours and then processed for bulk-RNA sequencing. Differential gene expression analysis revealed 43 up- and 40 downregulated genes in WLWH compared to MLWH upon IMQ stimulation. **(b)** Principal component analysis of top 5000 genes between WLWH and MLWH. **(c)** Differentially expressed genes in WLWH compared to MLWH upon IMQ stimulation based on FDR corrected p-values >0.05 and log2FoldChange. Genes from the X- and Y-chromosomes were excluded in this visualisation but can be found in the supplementary information. **(d)** Pathway enrichment analysis based on differentially expressed genes in WWH compared to MWH upon IMQ stimulation using the HALLMARK gene set.

We compared the transcriptomic signature of PBMCs from WLWH compared to MLWH upon IMQ stimulation. Principal component analysis suggested that sex was a major driver of variance in the IMQ-induced transcriptomic landscape, as PCs were only partially overlapping between WLWH and MLWH (**Figure 3a**). In total, 43 genes were upregulated and 40 downregulated in WLWH compared to MLWH (**Figure 3b**). IMQ−stimulated PBMCs from WLWH exhibited a broad interferon signature compared to MLWH, with increased expression of *IRF7* and multiple downstream interferon−stimulated genes (*MX1, OAS1, OAS3, IFIT1, IFIT3, IFI44, IFI44L*), as well as *RSAD2, ISG15*, and *USP18*. The RNA sensor *LY6E*, lectin *SIGLEC1*, and the RNA-editing enzyme *APOBEC3A* were also more strongly expressed in WLWH, indicating a multi-layered antiviral defence (**Figure 3c**). Additional female-biased transcripts included chemokine *XCL2*, antigen-presentation gene *HLA-DQB2*, and several X-linked genes known to escape XCI, such as *KDM5C*, *KDM6A*, and *STS* (**Supplementary Figures 4a, b**). These genes are involved in epigenetic regulation and steroid metabolism, suggesting enhanced immune activation and chromatin remodelling in WLWH. As expected, Y-linked transcripts were markedly under-represented in WLWH, including *KDM5D*, *DDX3Y*, *ZFY*, *UTY*, *EIF1AY*, *RPS4Y1*, and *USP9Y*, which are involved in transcriptional regulation, translation, and chromatin modification (**Supplementary Figures 4a, b**). In parallel, several autosomal genes associated with antibacterial and inflammatory responses were also downregulated in WLWH, notably *LTF*, *BPI*, *DEFA3*, *LCN2*, *OLFM4*, *MMP8*, and *ARG1*, as well as neutrophil activation markers *CLEC4D*, *CKLF*, and *PADI4*, suggesting a reduced neutrophil-mediated immune profile.

Consistent with transcript-level findings, gene set enrichment analysis (GSEA) revealed that WLWH upregulated both the HALLMARK_Interferon_Gamma_Response (FDR = 1.19×10^-19^) and HALLMARK_Interferon_Alpha_Response (FDR_j_=1.19×10^-19^) signatures (**Figure 3d**). KEGG pathway analysis further highlighted the female-biased enrichment of viral infection signatures (Hepatitis C, Influenza A, Measles, COVID-19, Epstein–Barr virus infection), while Gene ontology (GO) terms associated to antiviral defence (defence response to virus, type I interferon signaling, negative regulation of viral genome replication) were markedly induced. Conversely, antibacterial and inflammatory processes (defence response to bacterium, cellular response to lipopolysaccharide, TNF production) were downregulated (**Supplementary Figures 4c, d**), indicating a female−specific re-prioritization toward robust antiviral and interferon−mediated immunity in PLWH. Together, these genes represent coherent activation of the TLR7-MyD88-IRF7 signaling cascade, with IRF7 acting as the master transcriptional regulator driving downstream ISG expression, and APOBEC3A, LY6E, and SIGLEC1 functioning as additional innate restriction factors and sensors operating at multiple levels of the antiviral response.

**Figure 4:**
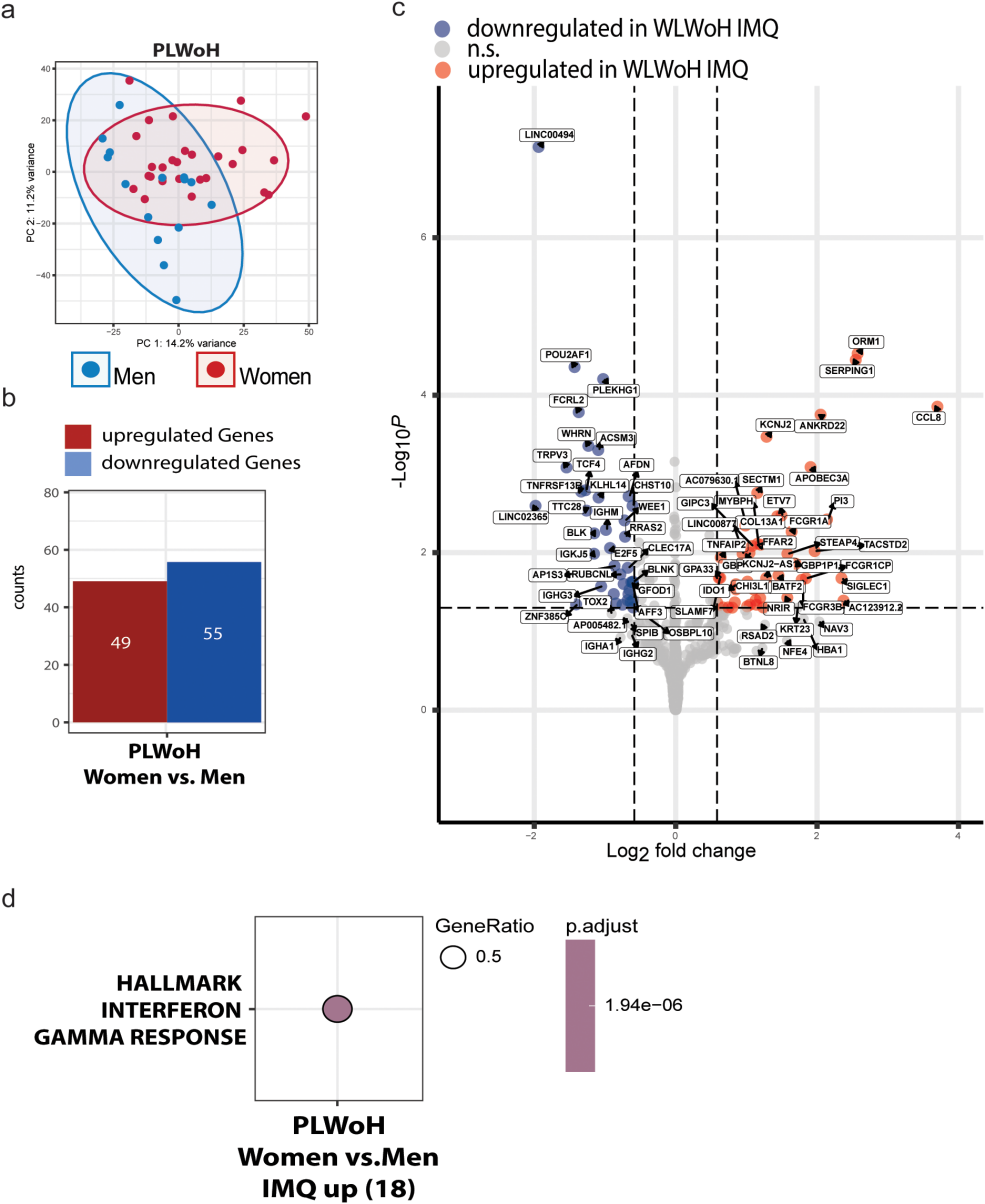
Transcriptomic signature of PLWoH reveals selective female−biased IFNγ responses in WLWoH. **(a)** PBMCs of 28 WLWoH and 15 MLWoH were stimulated with IMQ for 24 hours and then processed for bulk-RNA sequencing. Principal component analysis of top 5000 genes between women and men living without HIV. **(b)** Differential gene expression analysis revealed 49 up and 55 downregulated genes in WLWoH compared to MLWoH. **(c)** Differentially expressed genes in WLWoH compared to MLWoH upon IMQ stimulation based on FDR corrected p-values >0.05 and log2FoldChange. Genes from the X- and Y-chromosomes were excluded in this visualisation but can be found in the supplementary information. **(d)** Pathway enrichment analysis based on differentially expressed genes in WLWoH compared to MLWoH upon IMQ stimulation using the HALLMARK gene set.

Having observed sex-specific cytokine responses to TLR7 stimulation in PLWH, we next aimed to identify the underlying pathways and to disentangle whether these are general sex biases versus those unique to HIV infection. Therefore, we next analyzed IMQ-stimulated PBMCs from n=28 Women living without HIV (WLWoH) and n=15 men living without HIV (MLWoH). Principal component analysis again showed that sex was a dominant source of variation (**Figure 4a**). Overall, we found 49 upregulated genes and 55 downregulated (FDR < 0.05) in WLWoH compared to MLWoH (**Figure 4b**). WLWoH displayed significant upregulation of innate and antiviral effectors upon IMQ stimulation compared to men. Notably, classical viral restriction factors *IFITM3* and *APOBEC3A* were elevated, alongside the interferon−stimulated GTPase GBP1 and the lectin SIGLEC1 (**Figure 4c**). Chemokine *CCL8*, indoleamine−2,3−dioxygenase (*IDO1*), and the acute−phase inhibitor SERPING1 were also more strongly induced in MLWoH compared to WLWoH, reflecting a robust IFNγ–driven inflammatory milieu. As expected in female participants, depletion of Y−linked genes (e.g., *DDX3Y, ZFY, USP9Y, RPS4Y1, UTY*) (**Supplementary Figure 5b**) as well as key B−cell markers and signaling components (*CD19, BLNK, POU2AF1, FCRL2, BLK, CXCR5, TNFRSF13B*) were downregulated in WLWoH, as were transcription factors *BCL11A* and *PAX5* (**Supplementary Figures 5a, b**).

PBMCs of WLWoH were selectively enriched for the HALLMARK_Interferon_Gamma_Response (FDR = 1.94×10^-6^) (**Figure 4d**) but, in contrast to WLWH, showed a concurrent downregulation of B−cell–related pathways: both the KEGG “Primary immunodeficiency” and “B cell receptor signaling” pathways, as well as the GO BP “B cell receptor signaling” term, were significantly suppressed (FDR < 0.05) (**Supplementary Figures 5c, d**). This indicates a shift away from adaptive B−cell programs towards an increased IFNγ–driven innate profile in WLWoH, compared to MLWoH.

To confirm that the observed sex-specific ISG signature was driven by TLR7 activation rather than constitutive baseline differences, we analyzed bulk transcriptomic profiles of unstimulated (RPMI) PBMCs from the same donors. In the unstimulated condition, no interferon-stimulated genes were differentially expressed between WLWH and MLWH, nor between WLWoH and MLWoH (**Supplementary Figure 6**). While other sex-specific differences were present at baseline, the absence of a baseline ISG signature confirms that the robust interferon-associated transcriptional response we report is specifically induced by TLR7 activation rather than reflecting a pre-existing constitutive difference between sexes.

### 3.5 menopausal status modulates TLR7-mediated immune responses in women with HIV

Given the decline in estrogen and progesterone and the rise in follicle-stimulating hormone (FSH) during menopause (52), we investigated whether hormonal changes associated with menopausal status influence TLR7-mediated immune responses in WLWH. To isolate the effect of hormonal changes from age-related immune alterations, all analyses were corrected for age.

**Figure 5:**
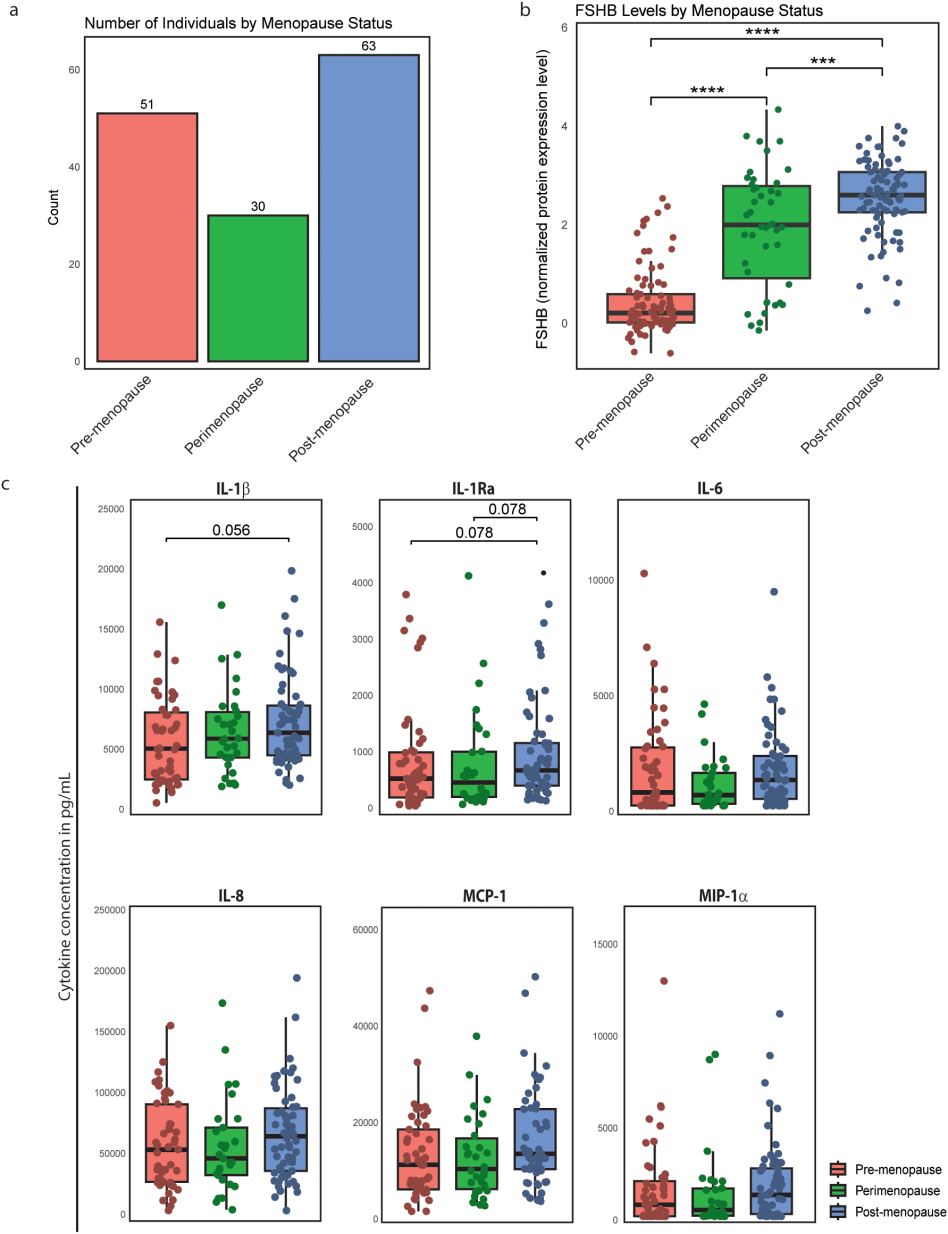
Impact of menopause status on cytokine production upon TLR7 stimulation in WLWH. **(a)** Number of WLWH categorised as pre-, peri- or post-menopausal according to self-reported questionnaire. **(b)** Plasma protein measurement of FSHB stratified for menopause status. **(c)** Cytokine production of PBMCs upon stimulation with IMQ stratified for menopause status. PBMCs were isolated and stimulated with IMQ for 24 hours. Cytokines between women living with HIV in different menopause stages were measured in the supernatant. Significance was tested using a logistic regression model, adjusted for age, season of inclusion, inclusion before or after COVID-19 pandemic, ancestry, and lymphocyte/monocyte ratio. P-values adjusted for multiple testing. Asterisks indicate statistical significance: ***P < 0.001, ****P < 0.0001.

We compared cytokine responses to IMQ stimulation in pre-menopausal (n=51), peri-menopausal (n=30), and post-menopausal (n=63) WLWH (**Figure 5a**), confirming self-reported menopausal status by measuring plasma FSHB concentrations (**Figure 5b**). Post-menopausal WLWH showed a trend toward higher IL-1β production compared to both pre-menopausal (p=0.078) and peri-menopausal WLWH (p=0.078). A similar trend was observed for IL-1Ra, with increased production in post-menopausal versus pre-menopausal WLWH (p=0.056) (**Figure 5c**), suggesting menopause-associated hormonal changes may enhance certain pro-inflammatory responses.

To assess whether these hormonal shifts also modulate TLR7-driven gene expression, we measured interferon-stimulated gene (ISG) expression in a subset of pre- (n=10), peri- (n=4), and post-menopausal (n=4) WLWH (**Supplementary Figure 7a**). While most ISGs did not differ significantly between groups, OLFM2 expression was significantly elevated in peri-menopausal (p=0.036) and post-menopausal WLWH (p=0.014) compared to pre-menopausal WLWH (**Supplementary Figure 7b**).

### 3.6 Sex, not HIV controller status, drives interferon responses following TLR7 stimulation

**Figure 6:**
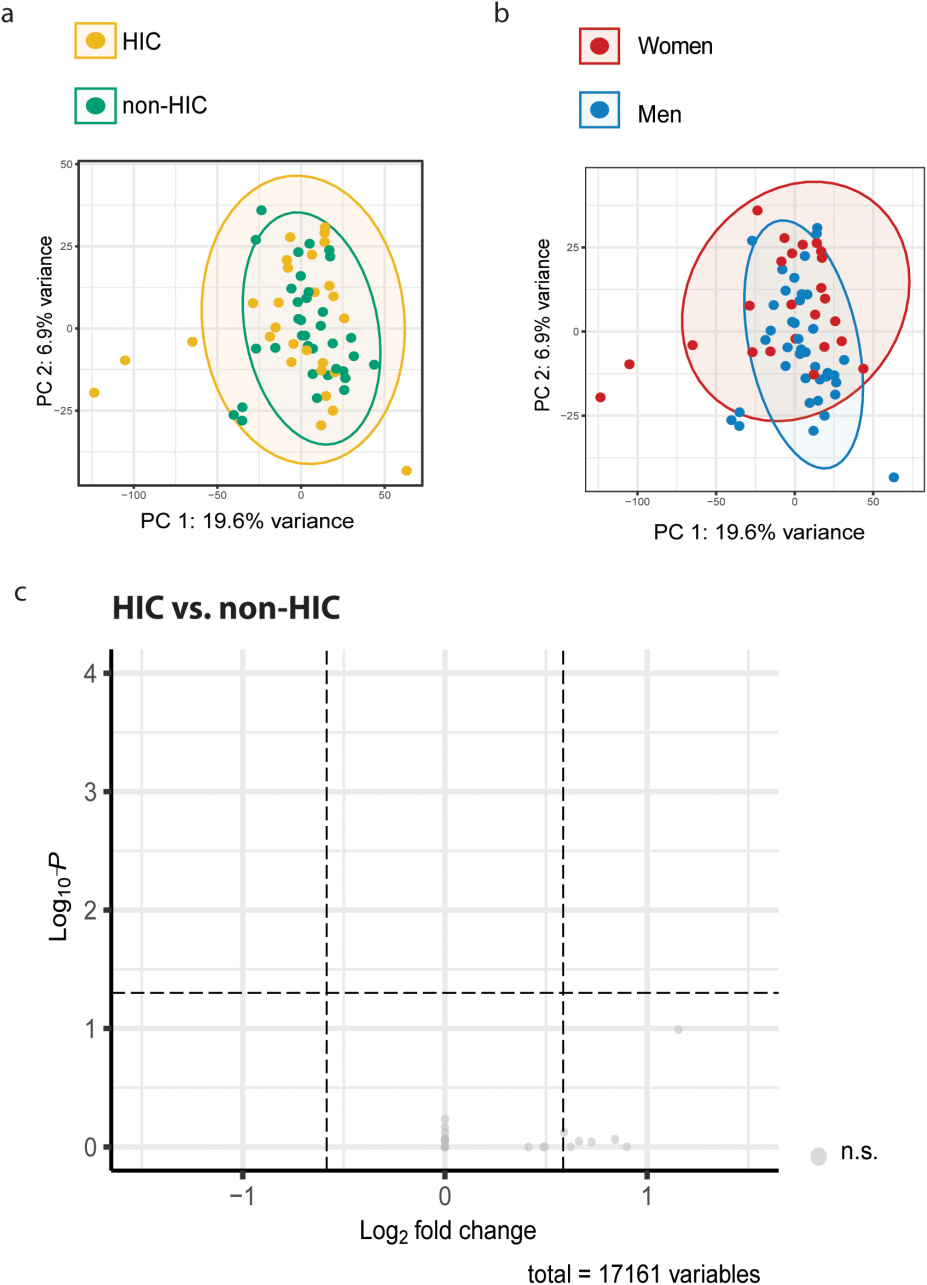
Differential Gene Expression of PBMCs of HIC vs. non-HIC upon TLR7 Stimulation. **(a)** PCA analysis of gene expression levels based on HIV controller status and **(b)** sex. **(c)** PBMCs of 28 HIC and 35 non-HIC were stimulated with IMQ for 24 hours and then processed for bulk-RNA sequencing. Differential gene expression analysis revealed that no genes were up- or downregulated upon IMQ stimulation in HIC compared to non-HIC.

To investigate whether the robust interferon-stimulated gene (ISG) signature observed in WLWH was specific for HIC, we performed bulk transcriptomic analysis on IMQ-stimulated PBMCs from 28 HIC and 35 non-HIC. These groups were matched for age and sex and did not significantly differ by ethnicity, season of inclusion, or COVID-19 vaccination status. PCA of gene expression showed substantial overlap between HIC and non-HIC (**Figure 6a**), whereas PCA based on sex revealed a more pronounced separation (**Figure 6b**). This was confirmed when performing DEG analysis, as no differentially expressed genes were identified between HIC and non-HIC (**Figure 6c**). This effect extended as well when comparing ART-naïve ECs (n=14) to non-HIC (**Supplementary Figure 8a**). This suggests that sex has a stronger impact on transcriptional responses to TLR7 stimulation than HIV controller status.

To confirm that the previously observed IFN-dominated transcriptional response was driven by sex and not by controller status, we repeated the analysis, excluding HIC and compared gene expression in 13 WLWH and 22 MLWH that were non-HIC. This analysis identified 35 upregulated and 29 downregulated genes in WLWH versus MLWH (**Supplementary Figure 8b**). Notably, upregulated genes in women included canonical ISGs such as *IRF7*, *HELZ2*, and *ISG15*, mirroring the transcriptional pattern observed in the full cohort (**Supplementary Figure 8c**). Pathway enrichment analysis again revealed significant induction of the HALLMARK gene sets “Interferon Alpha Response” and “Interferon Gamma Response” in women (**Supplementary Figure 8d**). These findings collectively indicate that the enhanced interferon signature and associated antiviral transcriptional response are predominantly driven by female sex rather than HIV controller status.

## 4 Discussion

This study reveals substantial sex-based differences in immune responses to TLR7 stimulation, particularly in the context of HIV infection. We demonstrate that women living with HIV exhibit a distinct immunological profile characterized by lower pro-inflammatory cytokine production and enhanced expression of ISGs following TLR7 activation. Some of these effects were specific for PLWH and were independent of pDC abundance or HIV controller status, indicating intrinsic cellular differences.

WLWH produced significantly lower IMQ-induced IL-1β and MIP-1α compared to MLWH, with trends toward lower IL-8 and IL-1Ra, consistent with a sex-specific skewing of TLR7 signaling away from pro-inflammatory cytokine production. These differences persisted after correcting for monocyte numbers, suggesting intrinsic sex-linked differences in cellular responsiveness rather than immune cell composition. Our findings align with previous studies demonstrating sex-based disparities in cytokine production, where WLWH produce less IL-6, IL-1β, and TNF upon TLR2/6 stimulation, and women generally show reduced IL-1β, IL-6, IL-8, IL-10, and TNF upon TLR7/8 stimulation (53). Lower monocyte counts in females are a well-established finding across both HIV-infected and healthy populations (89, 47) and Beenakker et al. demonstrated in a large pooled analysis that sex differences in monocyte-derived cytokine responses to innate stimulation largely disappear after correcting for monocyte concentration (90), supporting the interpretation that the reduced cytokine output in WLWH reflects both lower monocyte numbers and intrinsic differences in cellular responsiveness.

Estrogen, in particular E2, has been shown to modulate immune responses by suppressing pro-inflammatory cytokine production, such as IL-1β and TNF in monocytes (54, 55). Although our comparison between pre- and postmenopausal WLWH did not reach statistical significance, we observed a trend towards higher inflammatory cytokine levels in postmenopausal WLWH. Menopause is associated with an increase in inflammation and the development of chronic conditions, in an estrogen-dependent manner (56–58). It is suggested that this is a consequence of a decrease in estrogen and the subsequent increase in inflammatory cytokines, which is in alignment with our findings (59). Investigating the increase in chronic, inflammatory conditions in postmenopausal WLWH could further inform this line of research, but is out of the scope of this current manuscript.

In contrast to reduced inflammatory cytokines, PBMCs from WLWH exhibited a markedly enhanced interferon-driven transcriptional program upon TLR7 stimulation. IFNα concentrations in cell supernatants were below the detection limit at 24 hours, consistent with the well-established kinetics of pDC-derived type I IFN production, which peaks within 4–8 hours of TLR7 activation and declines substantially thereafter, strong upregulation of canonical ISGs such as IRF7, ISG15, MX1, OAS1, OAS3, IFIT1, IFIT3, RSAD2, and USP18, along with enrichment of both HALLMARK_Interferon_Alpha_Response and HALLMARK_Interferon_Gamma_Response gene sets, strongly suggests a coordinated type I interferon response in the hours preceding our transcriptomic readout. This suggests a broad and coordinated activation of IRF-driven interferon-associated transcriptional programs in WLWH. Given the well-documented overlap in target genes between type I and type II IFN pathways, and the established role of TLR7 in primarily driving type I IFN production, the enrichment of both Hallmark gene sets most likely reflects the breadth and magnitude of type I IFN-driven ISG induction rather than parallel activation of independent type I and type II IFN signaling cascades. This robust antiviral gene expression signature was further supported by elevated expression of immune effectors such as *APOBEC3A, SIGLEC1*, and *LY6E*, which function at various levels of viral restriction and sensing (60–62).

These findings are consistent with prior studies demonstrating sex-based differences in interferon responses. For example, Meier et al. showed that plasmacytoid dendritic cells (pDCs) from women produced more IFNα upon TLR7 stimulation using HIV-derived ligands, enhancing CD8^+^ T cell activation (27). While IFNα levels in the cell supernatant were below the detection limit, the robust induction of ISGs and interferon-related gene sets in PBMCs from WLWH supports the idea of increased interferon pathway activation downstream of TLR7. Similarly, Chang et al. reported increased ISG expression in female-derived cells in HIV infection, even after adjusting for viral load, with links to greater immune activation (63). Notably, type-I IFN responses can be pro- or anti-inflammatory, depending on the disease context and timing, as summarized by McNab et al. (64). Interestingly, type-I interferons such as IFNα have been shown to inhibit the NLRP3 inflammasome (65) and the production of IL-1β and IL-1α (66, 67). The elevated type-I interferon response in WLWH upon TLR7 stimulation, therefore, might be involved in the decreased production of inflammatory cytokines.

In addition, our baseline transcriptional profiling suggests that pDCs of WLWH are intrinsically “primed” for IFN-I responses. In unstimulated PBMCs, *IRF7* transcripts in pDCs were significantly higher in WLWH than in MLWH, whereas TLR7 expression and pDC abundance were comparable. This indicates that female pDCs maintain a higher basal activation of the IFN-I pathway. Our findings are in line with O’Brien et al. showing that pDCs from PLWH are known to constitutively secrete low amounts of type I IFN, which drives elevated baseline *IRF7* and ISG expression (68). Thus, the higher resting *IRF7* levels in WLWH likely reflect enhanced transcriptional priming of the interferon axis, predisposing their cells to the robust ISG induction which we have observed upon TLR7 stimulation. These findings reinforce the concept of sex-specific innate priming, whereby the IFN-I pathway is pre-activated at baseline in female pDCs, even in the absence of differences in pDC frequency or maturity. Beyond IRF7, our findings point to broader engagement of the TLR7 downstream signaling network in WLWH. TLR7 signals through MyD88 and IRAK4 to activate both NF-κB, driving pro-inflammatory cytokine production, and IRF7, which is the master regulator of type I IFN gene expression. (91) The observation that WLWH show simultaneously reduced NF-κB-driven cytokine outputs (IL-1β, MIP-1α) and enhanced IRF7-driven ISG induction suggests a sex-specific skewing of TLR7 signaling away from the inflammatory NF-κB branch and toward the antiviral IRF7-IFN branch. This division of TLR7 signaling has been observed in other contexts and may indicate differences in upstream regulatory factors, including the known inhibitory effect of type I IFNs on NF-κB-driven inflammasome activation and IL-1β production. (65) Additionally, the elevated expression of innate restriction factors APOBEC3A, LY6E, and SIGLEC1 in WLWH indicates engagement of multiple layers of the antiviral innate immune response beyond canonical IFN signaling, suggesting a broad and coordinated sex-specific antiviral program downstream of TLR7 activation. (60–62). Importantly, the absence of a sex-specific ISG signature in unstimulated PBMCs at baseline indicates that the enhanced interferon-associated transcriptional response in WLWH reflects a TLR7-activation-dependent program rather than a constitutive difference in IFN pathway activity, further supporting the specificity of TLR7-driven innate immune priming in women living with HIV.

Notably, in WLWoH only the HALLMARK_Interferon_Gamma_Response pathway was enriched, whereas in WLWH both HALLMARK_Interferon_Alpha_Response and HALLMARK_Interferon_Gamma_Response pathways were significantly upregulated. This differential pattern suggests that HIV infection broadens the sex-specific interferon-associated transcriptional response to TLR7 stimulation, engaging a wider IRF-driven ISG network beyond what is observed in uninfected women. Given the substantial overlap in target genes between these two Hallmark gene sets through shared IRF-mediated transcriptional mechanisms, this difference likely reflects the overall magnitude and breadth of ISG induction rather than selective activation of distinct type I versus type II IFN signaling cascades per se. This distinction is further supported by the absence of a sex difference in IFNγ protein production in either group, suggesting that the transcriptomic enrichment of interferon gamma response pathways reflects shared downstream ISG targets rather than differential IFNγ secretion. Our results are reflected in immune adaptations seen in other viral infections. For example, Cox et al. showed that HCMV infection in infants blunts TLR7/8 responses in women but not in men (69). In addition, recent research shows that pDCs of WLWH produced more IFNα and TNFα than those of uninfected women (25). While we did not make a direct comparison between people living with and without HIV, due to substantial confounding by ethnicity and the potential for HIV-induced immune alterations to overshadow TLR7-specific effects, our data clearly show that *Interferon Alpha Response* pathways are selectively enriched in PBMCs of WLWH. These studies suggest that viral infections intrinsically shape the response towards TLR7 activation in a substantial manner. Both studies linked increased TLR7 responses following viral infections to a trained immunity program, a concept describing long-term memory characteristics of innate immune cells via metabolic and epigenetic programming (70–72). The selective enrichment of type I IFN-associated transcriptional responses in WLWH but not WLWoH is consistent with this model. While trained immunity has been primarily studied in monocytes and macrophages (73, 74), NK cells also exhibit features of innate immune memory. Indeed, we found evidence for epigenetic remodelling of NK cells being associated with viral control and enhanced responsiveness upon restimualtion (36).

Beyond interferon-driven antiviral immunity, our transcriptomic analysis also revealed increased expression of several X-linked genes with established roles in epigenetic regulation in PBMCs from WLWH. Notably, *KDM5C* and *KDM6A*, which encode histone demethylases targeting H3K4me3 and H3K27me3 respectively, are well-established escape mechanisms from X-chromosome inactivation, have been implicated in modulating chromatin accessibility and gene expression in immune cells, and are involved in epigenetic modification of IRF4 and IRF5 (75–77). Elevated expression of these genes suggests that female PBMCs may have enhanced capacity for epigenetic remodelling, which could amplify or stabilize immune gene transcription following TLR7 stimulation. In addition, we observed upregulation of *STS* and *PUDP*, which also map to the X chromosome and are involved in steroid metabolism and nucleotide processing, respectively (“HDHD1, which is often deleted in X-linked ichthyosis, encodes a pseudouridine-5′-phosphatase Biochemical Journal Portland Press,” n.d.; 78). Their contribution to immune function is less well-characterized, and the expression may reflect broader sex-specific regulatory mechanisms. In conclusion, these findings raise the possibility that X-inactivation escape of epigenetic modulators contributes to transcriptional plasticity and enhanced antiviral responses in women, potentially reinforcing interferon-driven gene programs and shaping long-term immune activation profiles in WLWH.

Notably, we found no sex differences in pDC frequency, indicating that functional rather than numerical differences underlie the sex-biased interferon response. This is consistent with Berghöfer et al., who reported equivalent pDC numbers between sexes, yet greater IFNα production in women upon TLR7 stimulation (22). Interestingly, unstimulated pDCs from WLWH displayed higher basal *IRF7* transcripts than those from men (while *TLR7* expression was comparable). This suggests that female derived pDCs are intrinsically “pre-activated” in the IFN-I pathway. Indeed, Griesbeck et al. showed that higher resting IRF5 (another IFN-regulating factor) in female derived pDCs correlates with greater IFN-α production (79). While IRF7 itself is autosomal, its regulation in female pDCs may involve sex-specific factors. For instance, recent work by Schloer et al. identified DDX3 (an X-linked RNA helicase) as critical for sex-biased IFN responses: female pDCs had higher DDX3 expression, and DDX3 translocated with IRF7 to the nucleus to drive IFN-I gene expression (80). Similarly, the female advantage in IRF7 signaling may reflect the effects of estrogen receptors on IRF genes or faster IRF7 phosphorylation kinetics. Griesbeck et al. found that IRF7 activation occurs more rapidly in female pDCs (81). However, in our study, we did not see differences in women post-menopause, which is marked by a decrease in estrogen, which is likely due to a small sample size.

Individuals who naturally suppress HIV replication without antiretroviral therapy (ECs), have been shown to have functional pDCs capable of producing high levels of IFN-α and reducing HIV replication (82). This capacity is associated with reduced virus production and induction of T cell apoptosis, suggesting a role for type-I interferon responses in viral control. Moreover, studies have demonstrated that TLR agonists can enhance HIV-specific T cell responses by upregulation of HIV-1 restriction factors and IFN-α production by pDCs (83). These findings underscore the importance of pDC-derived type-I interferon-signaling in modulating immune responses against HIV. Strikingly, biological sex exerted a stronger influence on TLR7-mediated ISG expression than HIV controller status, with no transcriptional differences identified between HIC and non-HIC upon IMQ stimulation. This indicates that in the context of viral suppression intrinsic sex-linked factors are the dominant determinants of TLR7-driven immune responses. These findings have direct implications for TLR7-targeted HIV cure strategies, where sex should be considered as a key biological variable in therapeutic design and outcome evaluation.

The influence of ART on TLR7-driven responses appears limited under conditions of viral suppression. Consistent with Lester et al., who showed that ART normalizes the elevated TLR7 expression and responsiveness associated with untreated HIV infection (92). We observed no significant transcriptional differences upon TLR7 stimulation when comparing ART-naïve elite controllers to ART-treated non-controllers. While the modest number of ART-naïve individuals precludes a definitive conclusion, these data suggest that ART use per se does not substantially alter TLR7-driven immune responses once viral suppression is achieved.

While we cannot draw conclusions about individuals with uncontrolled HIV replication, our findings indicate that in chronic, suppressed infection, intrinsic sex-related factors such as hormonal influences and X-linked gene expression may play a dominant role in shaping TLR7-driven immune responses. These insights have important implications for the development of TLR7-targeted therapies. Given the potential for enhanced IFN signaling to facilitate reservoir disruption, as well as the risk of immune exhaustion and autoimmunity, it is crucial to consider sex as a biological variable in therapeutic strategies. Tailoring treatments to account for sex-based differences in TLR7 responses may improve efficacy and reduce adverse events. TLR7 agonists, such as GS-9620 (vesatolimod), are being explored as latency-reversing agents in HIV cure strategies. In chronic hepatitis B patients, vesatolimod induced dose-dependent ISG15 upregulation, and this pharmacodynamic marker correlated with female sex, suggesting that women may mount stronger ISG responses to TLR7 agonists (84). Two clinical studies have tested vesatolimod in PLWH but did not evaluate sex differences (85, 86). However, recently, a study by Holmberg et al. suggested immune cells of WLWH may have a more robust immune activation when treated with GS-9620 *ex vivo*, which is in-line with our findings (87). While enhanced IFN signaling could facilitate reservoir disruption, it may also elevate risks of TLR7-triggered autoimmunity or immune exhaustion, as chronic IFN type I exposure has been linked to accelerated CD4^+^ T cell decline and comorbidity burden in WLWH. Additionally, persistent activation is associated with immune exhaustion and a higher burden of non-AIDS comorbidities, which disproportionately affect women with HIV (88).

Future research should focus on elucidating the mechanisms underlying sex-based differences in TLR7-mediated immunity and their impact on HIV pathogenesis and treatment outcomes. Understanding these dynamics will be essential for optimizing therapeutic approaches and improving the health of people living with HIV. In summary, our study highlights significant sex-based differences in immune responses to TLR7 stimulation, with WLWH exhibiting reduced pro-inflammatory cytokine production and enhanced antiviral interferon responses. These findings have important implications for understanding sex-specific HIV pathogenesis and developing adapted therapeutic strategies targeting TLR7 pathways.

### 4.1 Limitations of the study

The present study provides convergent evidence from bulk cytokine assays and transcriptomic profiling that PBMCs of women living with HIV mount reduced pro-inflammatory cytokine responses yet a markedly enhanced interferon-stimulated gene program after TLR7 stimulation; these patterns remained after correction for monocyte numbers and were independent of pDC frequency, supporting the interpretation of intrinsic, sex-linked differences in cellular responsiveness. Nevertheless, several limitations should be considered when interpreting these results. We did not obtain single-cell transcriptional data after IMQ stimulation, which limits our ability to assign the stimulus-induced transcriptional programs to specific cell subsets and to resolve heterogeneity in cell-type responses.

A further important limitation is the male-biased composition of the 2000HIV cohort, which reflects the epidemiology of HIV in Western Countries, but results in substantially unequal group sizes (1,134 men vs 192 women). While all analyses were corrected for relevant confounders, and the consistency of findings across cytokine, bulk RNA-seq, and scRNA-seq platforms provides convergent evidence, the reduced number of women limits statistical power for female-specific subgroup analyses. Similarly, the healthy control cohort was small (43 individuals) and unequally distributed between sexes (28 women, 15 men), limiting the power of sex comparisons in HIV-negative individuals and preventing a well-powered direct comparison between PLWH and PLWoH. The substantial ethnic differences between male and female participants, with women being more ethnically diverse and having a higher proportion of sub-Saharan African ancestry, represent an additional source of potential confounding that statistical correction for genetic principal components can only partially mitigate. Together, these factors mean that while our findings strongly suggest HIV infection amplifies sex-specific TLR7-driven interferon responses, a definitive claim of HIV-specificity would require a larger, ethnically and demographically matched cohort of HIV-negative controls, which was beyond the scope of the current study. Additionally, autoimmune status was not assessed in the healthy control cohort, and given the higher prevalence of autoimmune disorders in females, subclinical autoimmunity cannot be excluded as a contributing factor to the observed suppression of B cell receptor signaling pathways in WLWoH.

Our observation of enhanced type-I IFN activity relies primarily on robust ISG induction rather than direct detection of secreted IFNs, as IFNα concentrations in supernatants were below the limit of detection, and IFNγ showed no sex difference in when measured in a small subset of individuals. Therefore, assay sensitivity and sample size constrain cytokine-level conclusions. Strong confounding by factors such as ethnicity prevented a well-powered, direct comparison between females with and without HIV, and the rare phenotype of spontaneous HIV controllers resulted in a small controller subgroup that limits the precision of controller-specific inferences. Importantly, despite these limitations, the consistency between cytokine readouts and transcriptomic signatures provides a robust signal of sex-biased TLR7 responsiveness in WLWH.

## Data Availability

The original contributions presented in the study are publicly available. This data can be found here: https://doi.org/10.34973/3r13-k726.
