# Supplementary material for: Enhanced TLR7-induced interferon responses in women living with HIV
Source: Front Immunol. 2026 Apr 21;17:1791579. doi: 10.3389/fimmu.2026.1791579 (PMC13139085; doi:10.3389/fimmu.2026.1791579)
Supplement: Supplementary file 1 [file Table1.docx]

## Supplementary Material

Supplementary Tables

**Table S1: Description of the antibodies in panel 1**

| Specificity | Fluorochrome | Clone | Cat. Number | Company | µl  per donor | Purpose |
| --- | --- | --- | --- | --- | --- | --- |
| CD3 | BUV395 | UCTH1 | 563546 | BD Biosciences | 5 | T-cell lineage |
| HLA-DR | BUV661 | G46-6 | 612980 | BD Biosciences | 5 | Activation marker |
| CD86* | BUV737 | FUN-1 | 612784 | BD Biosciences | 2 | Costimulatory in APCs |
| CD14/  TCRVd2 | PB | RM052/  IMMU389 | ---- | ---- | ---- | CD14: Monocyte marker/ TCRVd2: T cell receptor expression for variant d2 |
| CD45 | KrO | J33 | ---- | ---- | ---- | White blood cells lineage |
| CD11c* | BV605 | 3.9 | 301636 | BioLegend | 5 | Classical dendritic cells |
| CD11b | BV650 | ICRF44 | 301336 | BioLegend | 10 | Integrin marker, expressed mainly in innate cells |
| CD274  (PDL1) | BV786 | 29E.2A3 | 329736 | BioLegend | 5 | Constitutive, low levels expressed on resting lymphocytes and APCs. immunoregulatory PD-1/PD-L1 pathway is operative during a persistent viral infection in humans, and define a reversible defect in HIV-specific T-cell function. |
| CD16 | FITC | 3G8 | ---- | ---- | ---- | NK subsets distinction, neutrophils and monocytes subsets |
| CD8 | PerCP | RPA-T8 | 301030 | BioLegend | 1 | T-cell lineage |
| CD184  (CXCR4) | PE | 12G5 | ---- | ---- | ---- | Th marker |
| CD56 | ECD | N901NKH-1 | ---- | ---- | ---- | NK cell lineage/NKT-like cell |
| CD1c | PC5 | L161 | ---- | ---- | ---- | Myeloid dendritic cells. CD1c+ DCs are susceptible to HIV infection, enables viral antigen production but impairs their immune functions and survival. |
| CD40/  TCR PAN gd | PC5.5 | MAB89/IMMU510 | ---- | ---- | ---- | CD40: B cells, iDCs and follicular DCs/ CD40 signaling may regulate MBC subset development.  TCR PAN gd: gd T-cell lineage |
| CD123/  TCRVd1 | PC7 | SSDCLY107D2/R9.12 | ---- | ---- | ---- | Plasmocytoid DCs, Basophils/  T cell receptor expression for variant d1: important in intestinal barrier function, increase in frequency in HIV infected individuals, including HIV controllers. |
| CD195  (CCR5) | APC | J418F1 | 359122 | BioLegend | 2 | HIV co-receptor (T cells, NK cells and Monocytes) |
| CD19/  CD4 | AA700 | J3.119/13B8.2 | ---- | ---- | ---- | CD19: B-cell lineage /CD4: T-cell lineage |
| CD66b | AA750 | 80H3 | ---- | ---- | ---- | Neutrophils |
| ViaKrome | IR885/40 | ---- | C36628 | Beckman  Coulter | --- | Selection of viable cells |
| PBA  (1% BSA in PBS) | ---- | ---- | ---- | ---- | 42 | Washing cells |
| Brillian stain buffer | ---- | ---- | 563794 | BD Biosciences | 50 | Required for staining with polymer dyes (BV abs) |

Antibodies highlighted in blue were included as a dried formulation in the custom DURA Innovation tube. The rest of the antibodies were added as a “drop-in mix”.

*Antibodies used in a pre-staining step.

Supplementary Figures


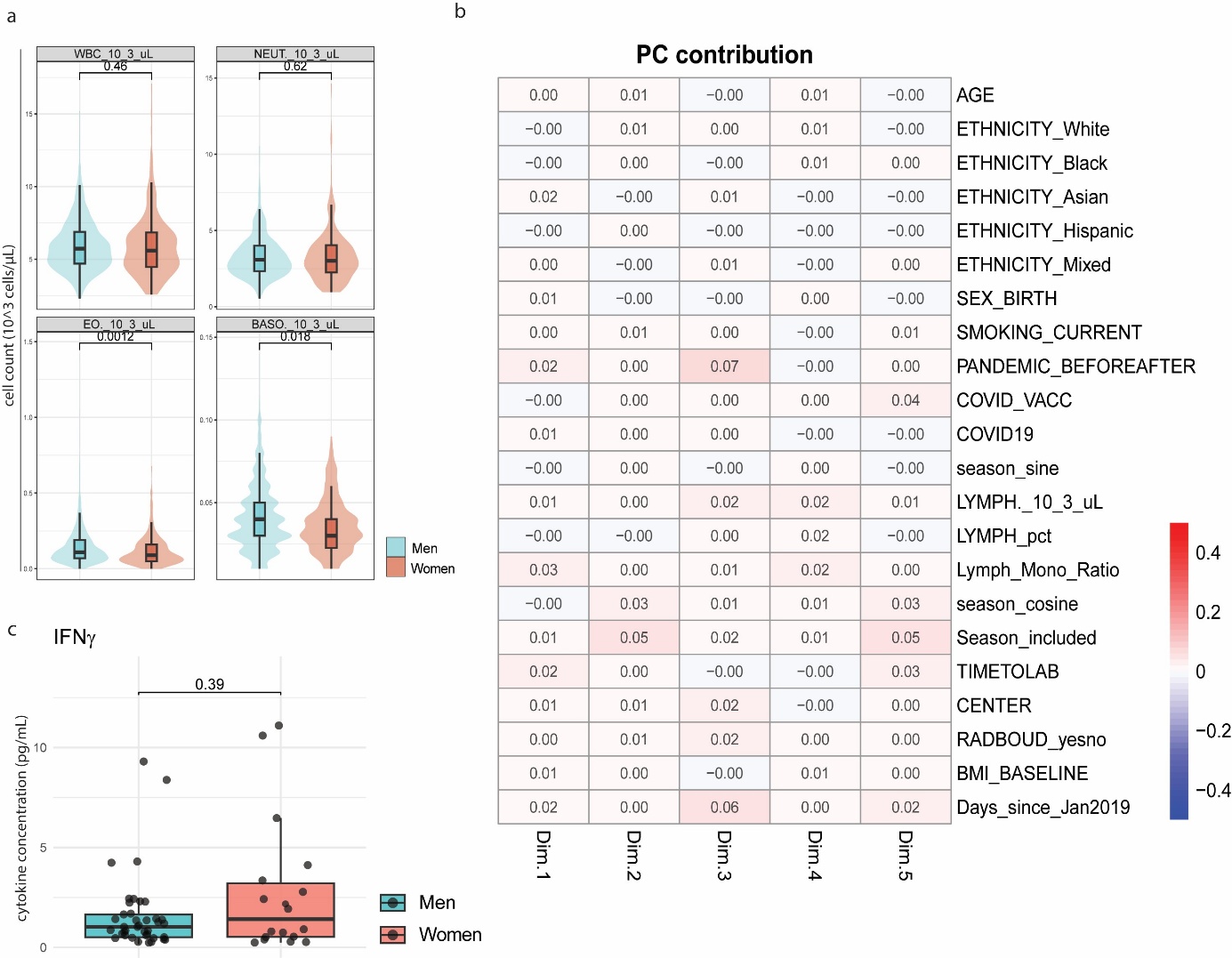


**Figure S1: Immune cell composition between men and women living with HIV and principal component analysis of PBMCs stimulation with IMQ.**

(a) Sysmex measurement data of all immune cells between women and men living with HIV. (b) Principal component analysis to determine confounders in cytokine production data. (d) Measurement of IFNγ in cell supernatant after PBMC stimulation with IMQ. Measurement of cell-supernatant of WLWH (n=18) and MLWH (n=41) of the 2000HIV-TRAINED study done using an Ella Automated Immunoassay.


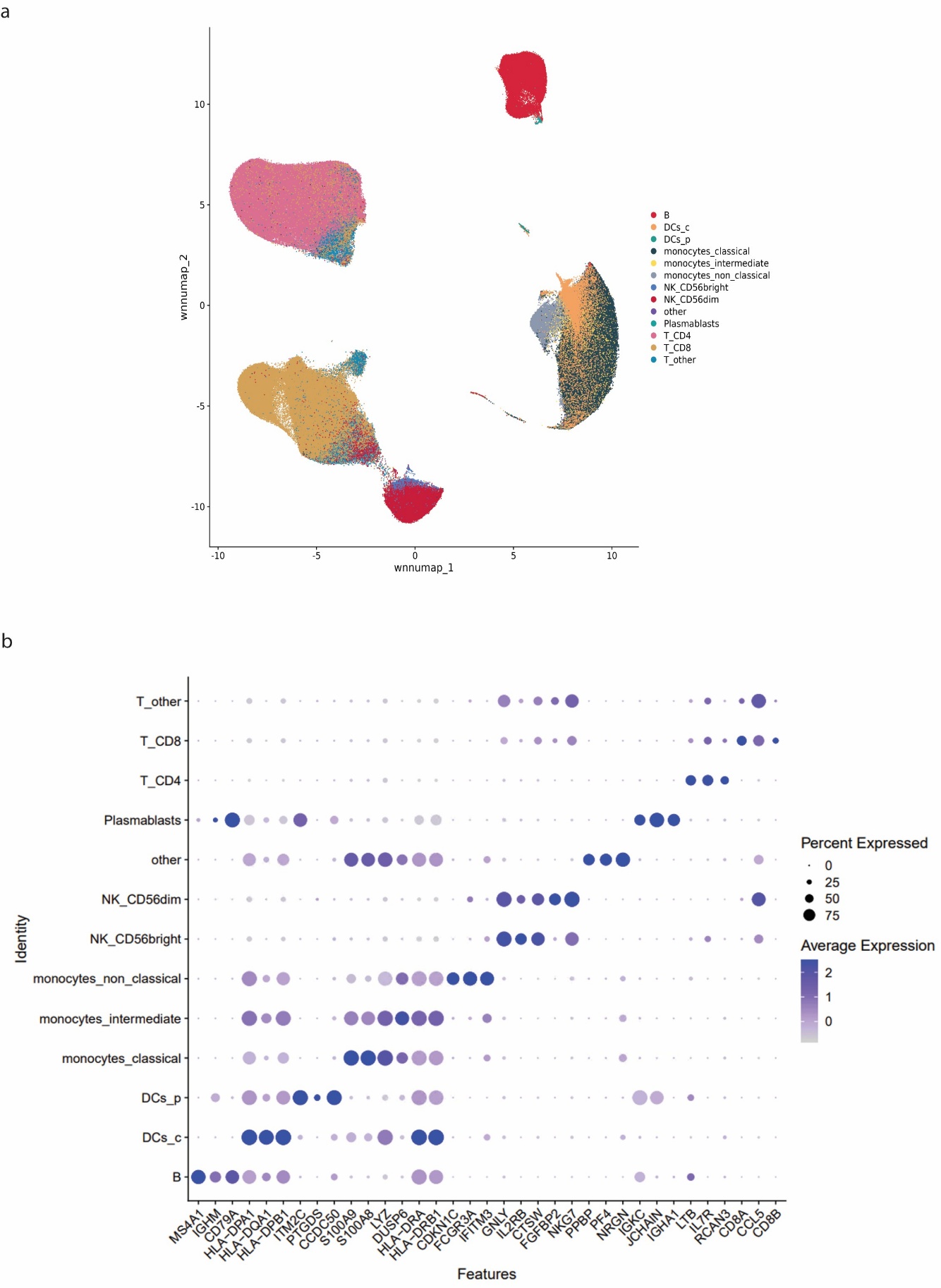


**Figure S2: scRNA sequencing of baseline PBMCs between women and men living with HIV**

(a) UMAP embedding of baseline PBMCs from PLWH, showing annotated immune cell populations. (b) Marker gene expression used to identify major immune cell types in the scRNA-seq dataset. The dot plot displays the expression levels and percentage of cells expressing key canonical genes across annotated cell clusters, enabling robust cell type classification.

**
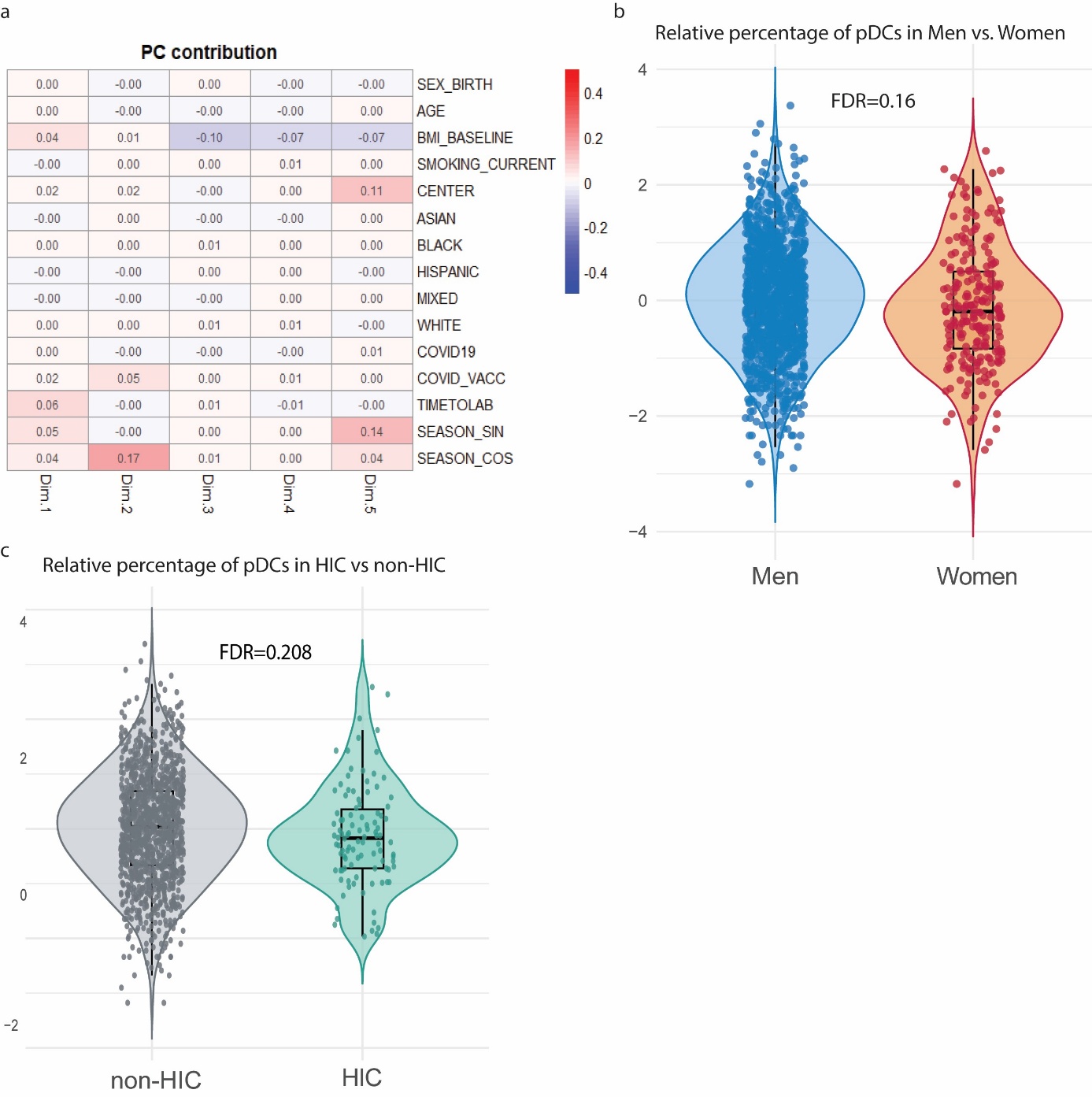
**

**Figure S3: Flow cytometry of pDCs in whole blood of women and men living with HIV**

(a) Principal component analysis of linear regression analysis of flow cytometry analysis. Relative percentages of pDCs between (b) men and women living with HIV and (c) n= 102 HIC and n = 1213 non-HIC


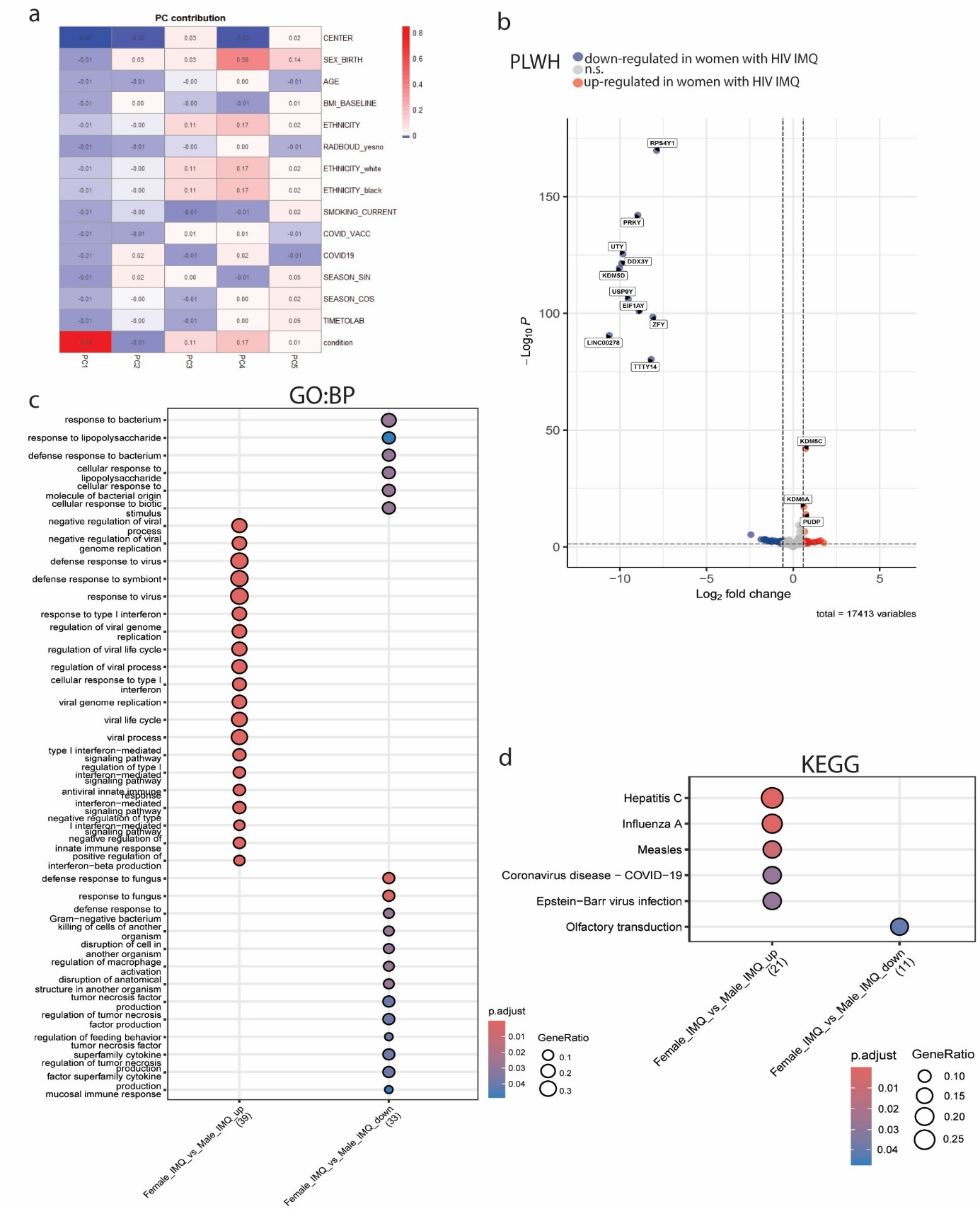


**Figure S4: Transcriptomics analysis of PBMCs stimulated with IMQ of PLWH and PLWoH**

(a) Principal component analysis to determine confounders in gene expression data between people living with HIV. (b) Volcanoplot of differentially expressed genes between women vs men living with HIV, including X- and Y-linked genes. (c) Pathway enrichment of differentially expressed genes in women vs men living with HIV using the GO:BP dataset and (d) the KEGG dataset.


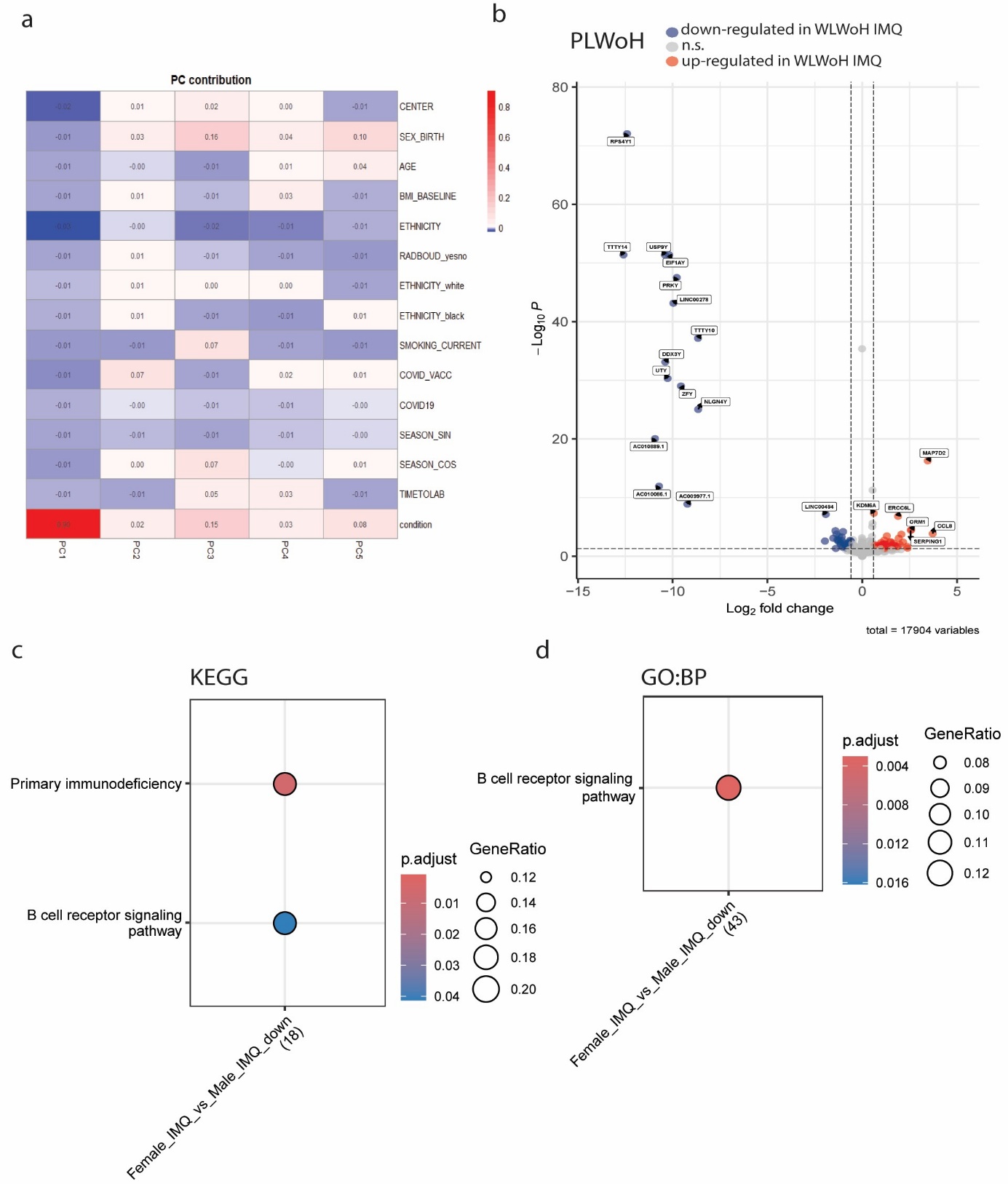


**Figure S5: Pathway enrichment of differentially expressed genes in women vs men living with and without HIV.**(a) Principal component analysis to determine confounders in gene expression data between people living without HIV. (b) Volcanoplot of differentially expressed genes between women vs men living without HIV, including X- and Y-linked genes. (c) Pathway enrichment of differentially expressed genes in women vs men living wihtout HIV using the KEGG dataset and (d) the GO:BP dataset.


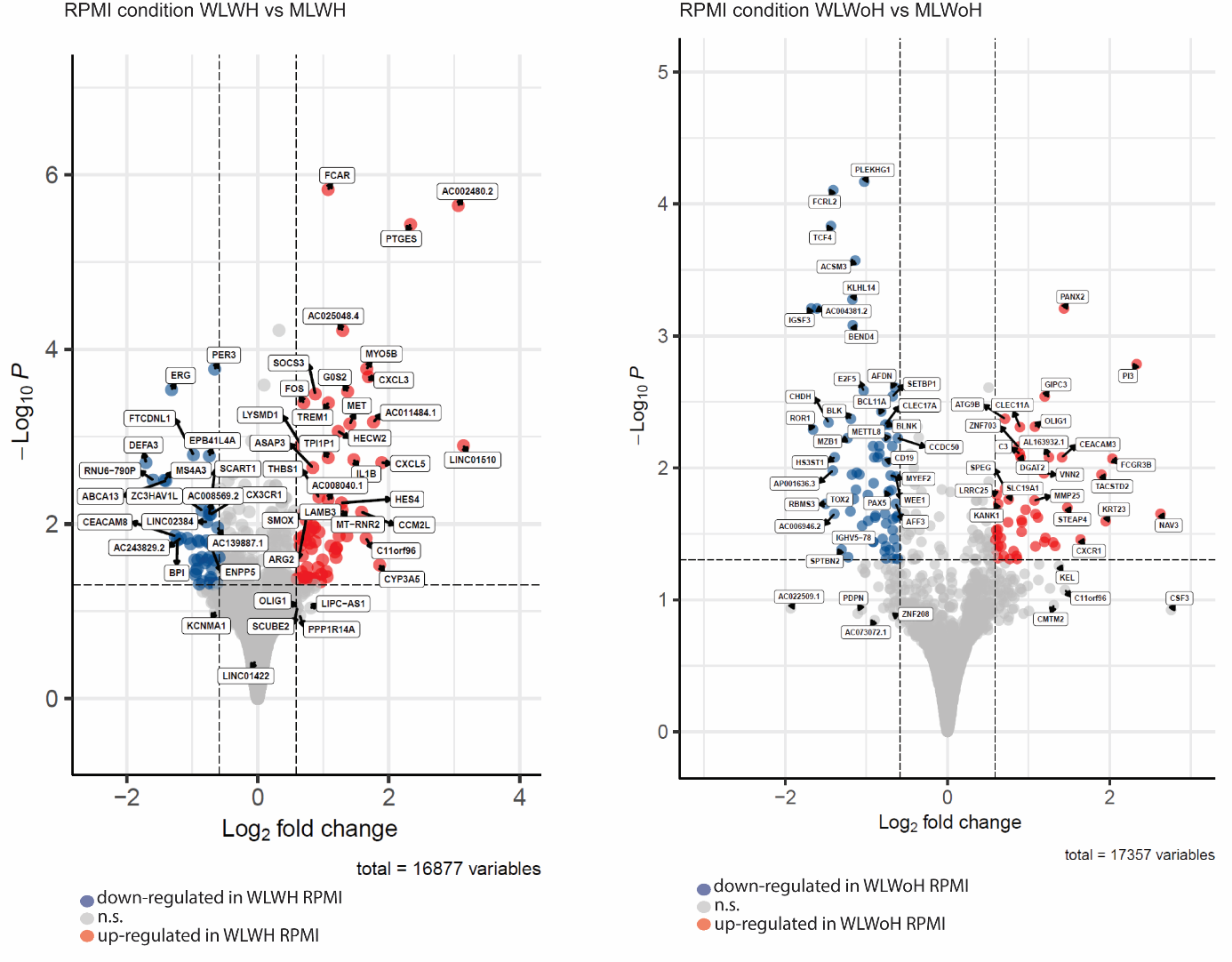


**Figure S6: Pathway enrichment of differentially expressed genes at baseline RPMI condition in women vs men living with and without HIV.**Volcano plot of differentially expressed genes between women and men living with HIV (left) and women and men living without HIV (right) at baseline unstimulated (RPMI) condition, excluding X- and Y-linked genes. No interferon-stimulated genes were differentially expressed between sexes in either group under unstimulated conditions.


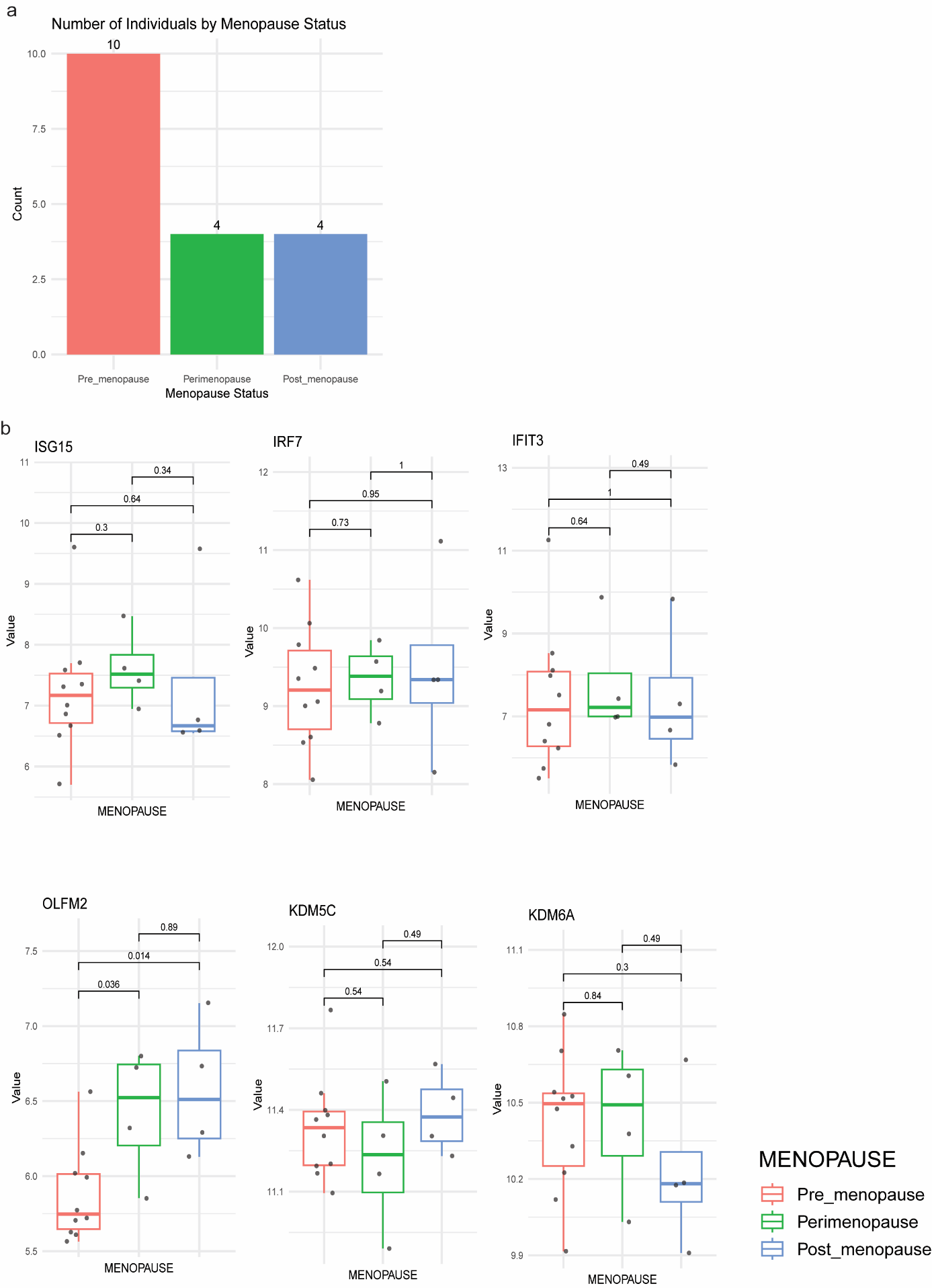


**Figure S7: Impact of menopause status on gene expression in women living with HIV upon IMQ stimulation.**

(a) Number of women categorised as pre-, peri- or post-menopausal in the bulk RNA sequencing. (b) Gene expression of selected ISGs in PBMCs upon stimulation with IMQ according to menopause status.

**
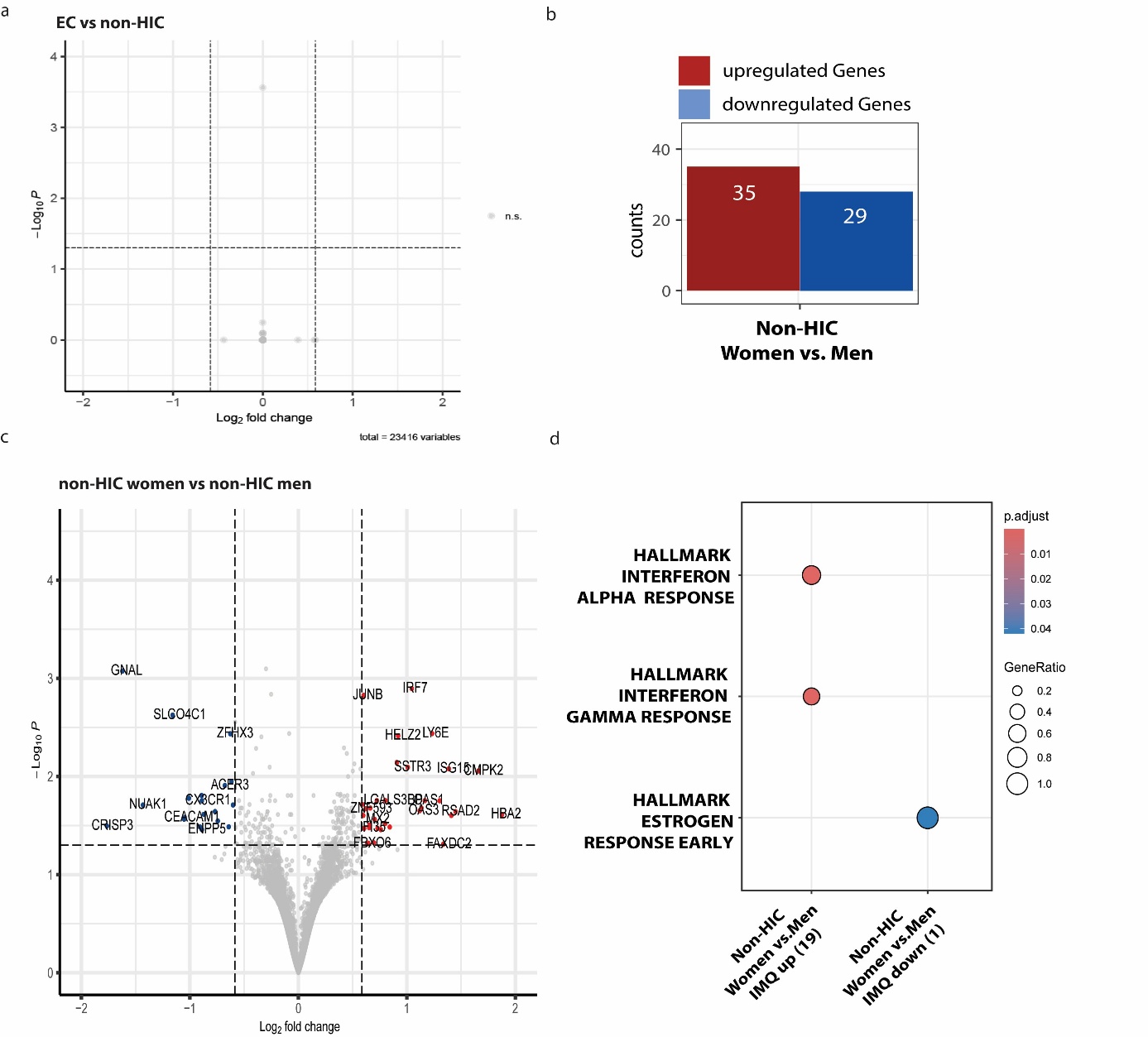
**

**Figure S8: Differential Gene Expression of ECs, and non-HIC women and men upon TLR7 stimulation**

(a) Differential gene expression between ECs and non-HICs revealed no genes were up- or downregulated upon TLR7 stimulation with IMQ. (b) Differential gene expression between non-HIC women and non-HIC men resulted in 35 upregulated and 29 downregulated genes upon IMQ stimulation. (c) Volcanoplot of up- and downregulated genes in non-HIC women compared to non-HIC men. (d) pathway enrichment of non-HIC women compared to non-HIC men upon IMQ stimulation.
